# Levetiracetam and Peripheral Oxidative Stress Biomarkers in Epilepsy: A Systematic Review of Human Evidence

**DOI:** 10.3390/ijms27188410

**Published:** 2026-09-21

**Authors:** Robert Mîndreanu, Simona Clichici, Bianca Buleteanu, Mario Badea, Mihaela Pop, Răzvan Mircea Cherecheș, Cezar Login

**Affiliations:** 1 Department of Physiology, Iuliu Hațieganu University of Medicine and Pharmacy, 400012 Cluj-Napoca, Romania; robert.flor.mindreanu@elearn.umfcluj.ro (R.M.); cezar.login@umfcluj.ro (C.L.); 2 Faculty of Medicine, Victor Babeș University of Medicine and Pharmacy, 300173 Timișoara, Romania; bianca.buleteanu@student.umft.ro (B.B.); mario.badea@student.umft.ro (M.B.); 3 Department of Public Law and Private Law, Faculty of Law and Economic Sciences Târgu Jiu, Titu Maiorescu University of Bucharest, 210102 Târgu Jiu, Romania; 4 HIVE Health Innovation Centre, Babeș-Bolyai University, 400132 Cluj-Napoca, Romania; razvan.chereches@ubbcluj.ro

**Keywords:** levetiracetam, epilepsy, oxidative stress, lipid peroxidation, glutathione, biomarkers, systematic review, SWiM, risk of bias, evidence gap

## Abstract

Oxidative stress is implicated in epilepsy, but seizures, disease severity, diet, comorbidity and treatment all influence circulating redox biomarkers. We systematically reviewed human evidence on levetiracetam (LEV) under three estimands: within-person change, LEV-treated epilepsy versus healthy controls, and LEV versus other antiseizure medications. PubMed/MEDLINE, Cochrane Library, Web of Science and Scopus were searched from inception to 15 July 2026 (PROSPERO CRD420261448016; OSF HXA4U). Eleven non-randomized studies contributed 297 participants—the sum of LEV subgroups with analysable biomarker data, not the total enrolled populations. Effect estimates with 95% confidence intervals were calculated here wherever reporting permitted; none could be derived for four studies, and those derived are wide, inconsistent in direction, or from designs that cannot attribute change to LEV. Meta-analysis was inappropriate: definitions, matrices, assays, estimands and variance reporting were incompatible. Certainty was very low for every outcome-domain/comparator body, and a sensitivity analysis excluding one report failing a post hoc source-verifiability check (10 studies, 265 participants) changed no conclusion. This review’s contribution is not an estimate of LEV’s redox effect but a demonstration of why the literature cannot establish one: the human evidence is not of sufficient validity to support or refute such an effect.

## 1. Introduction

Epilepsy is a chronic brain disorder characterized by an enduring predisposition to epileptic seizures and associated neurobiological, cognitive, psychological, and social consequences [1]. Oxidative stress has been implicated in epileptogenesis and seizure-related injury through mitochondrial dysfunction, excessive reactive oxygen and nitrogen species, lipid peroxidation, protein and nucleic-acid oxidation, and disruption of endogenous antioxidant systems [2,3]. The relationship is bidirectional: seizures can increase oxidative burden, while redox imbalance may increase neuronal excitability. An abnormal circulating biomarker in a treated patient may therefore reflect the disease, recent seizures, treatment response, diet, systemic inflammation, comorbidity or a medication effect. Levetiracetam is a widely used antiseizure medication whose principal pharmacological target is synaptic vesicle protein 2A (SV2A) [4]. Its limited cytochrome P450 metabolism and relatively low potential for pharmacokinetic interactions distinguish it from several older antiseizure medications. Experimental studies have also proposed anti-inflammatory, mitochondrial, and redox-modulating actions, but preclinical findings are not uniform and cannot be assumed to translate into a systemic antioxidant effect in humans [5,6,7].

Human investigations of LEV and oxidative status have used biomarkers that are biologically related but not interchangeable: products of molecular damage, antioxidant enzymes and composite measures, low-molecular-weight compounds, and, more recently, ferroptosis-related measures. Malondialdehyde measured by thiobarbituric-acid-reactive methods has limited analytical specificity, whereas F2-isoprostanes provide a more chemically specific index of lipid peroxidation [8,9]; and enzyme activity may rise as a compensatory response or fall through depletion, so isolated enzyme values cannot be interpreted without the broader pattern.

The primary studies also address different estimands. A before-after comparison asks whether biomarkers change over time; a comparison with healthy volunteers combines treatment status with the presence of epilepsy; a comparison with another antiseizure medication estimates a relative contrast subject to confounding by indication. Combining these designs into one pooled or directional summary would obscure rather than resolve the distinctions.

We therefore conducted a systematic review and structured synthesis of human LEV evidence with three objectives: (1) to characterize LEV-relevant findings by biomarker domain and comparator estimand; (2) to determine whether quantitatively compatible evidence exists for meta-analysis, and to calculate individual effect estimates wherever source reporting permitted; and (3) to evaluate how risk of bias, measurement limitations and clinical heterogeneity affect certainty and interpretation. This review does not produce an estimate of the redox effect of LEV, because the primary literature does not support one; its contribution is to identify, at the level of the individual biomarker result, which features of that literature prevent the causal question from being answered.

## 2. Methods

### 2.1. Protocol, Registration, and Reporting

The protocol was registered prospectively in PROSPERO (CRD420261448016) before study selection and deposited on the Open Science Framework (OSF; https://doi.org/10.17605/OSF.IO/HXA4U, accessed on 10 September 2026). Reporting follows PRISMA 2020 and the Synthesis Without Meta-analysis (SWiM) guideline [10,11]. No material deviation from the prespecified eligibility criteria occurred. Two included studies contributed results from matrices beyond those listed in the protocol (Section 2.5). The decision not to pool followed the prespecified provision for structured synthesis when pooling is inappropriate. Two additions were made after registration and are identified as post hoc throughout: the source-verifiability and reporting-consistency check of Section 2.7, and the sensitivity analysis of Section 3.9 that follows from it.

### 2.2. Eligibility Criteria

We included human studies enrolling people who received LEV for any underlying condition, with epilepsy as the anticipated primary population, reporting at least one oxidative stress, antioxidant, redox-related or ferroptosis-related outcome identifiable for a LEV group or treatment period. Eligibility was deliberately not restricted to epilepsy at the protocol stage: LEV is prescribed outside epilepsy, and narrowing the population in advance to the setting in which an effect was anticipated would have selected the evidence base on the expected result. That no study of another condition proved eligible is therefore a finding rather than a design choice. Eligible designs included uncontrolled before-after, non-randomized intervention, cohort, cross-sectional and case–control studies; eligible comparators were pretreatment baseline, healthy participants, or another active antiseizure medication.

We excluded animal, in vitro, and ex vivo studies; reviews and non-primary publications; case reports or case series with fewer than five participants; studies in which LEV was administered to healthy volunteers without an underlying condition; studies without an eligible biomarker; and studies in which LEV exposure or biomarker results could not be separated from pooled multidrug or drug-class data. Studies of supplements or other interventions in populations that happened to include LEV users were excluded unless they provided an estimable LEV effect.

### 2.3. Information Sources and Search Strategy

PubMed/MEDLINE, the Cochrane Library, Web of Science Core Collection and Scopus were searched from inception through 15 July 2026. Strategies combined controlled vocabulary, where available, with free-text synonyms for LEV and terms for oxidative stress, reactive oxygen or nitrogen species, lipid peroxidation, antioxidant enzymes, glutathione, DNA oxidation, isoprostanes, total antioxidant or oxidant status, aminothiols and related biomarkers. No date restriction was applied. Complete strategies are in Supplementary Table S1.

Embase was not searched: no institutional or individual access was available within the review period, and documentation can be supplied on request. We do not claim that Scopus and Web of Science compensate, and this review’s own coverage data argue against such a claim—no single database retrieved more than 9 of the 11 included studies, and three were retrieved by one database only (Supplementary Table S7). The omission is therefore material rather than formal and is treated as such in Section 4.6. Trial registries, WHO ICTRP, dissertations, preprints and other grey literature were not systematically searched. The restriction to English-language full-text articles was prespecified in the registered protocol; language bias is discussed in Section 4.6. Supplementary searching comprised screening the reference lists of all included studies and of the reviews retrieved electronically, plus forward-citation checking of the included studies in Scopus and Web of Science on 15 July 2026.

### 2.4. Study Selection and Data Extraction

Two reviewers independently screened titles and abstracts and assessed potentially eligible full texts. Data were extracted independently using a piloted form and reconciled by consensus after rechecking the source; unresolved disagreements went to a third reviewer. Captured items were design, setting, population, age, LEV sample size, dose, duration, comparator, seizure and treatment context, matrix, assay, biomarker values, dispersion, statistical results, attrition, and factors bearing on confounding or measurement validity.

No summary measure was transformed. Values appear in the metric used by the source—means with standard deviations, means with standard errors (identified as such), or medians with interquartile ranges. Medians were not converted to means, standard errors were not converted to standard deviations, and graphically presented results were recorded as figure-only rather than estimated from figures. Study authors were not contacted, including those of the report discussed in Section 3.4.

Two records were identified as linked to an included report rather than independent studies—a 2009 report and a 2017 trial-registry entry, each linked to a different included study. Neither was treated as a duplicate on title similarity alone: each was retrieved in full, compared with its primary publication for participants, outcomes or time points not present there, and then excluded as a companion record (Supplementary Note S3).

### 2.5. Outcome Framework

The synthesis unit was each biomarker result within a defined comparison, with direction assigned from the observed estimate rather than from statistical significance. Multiple results from the same cohort—several biomarkers, doses or follow-up times—were never aggregated into a study-level value and never contributed more than one result to a single biomarker-by-comparator cell; where several follow-up times were reported for one biomarker, the prespecified primary time point was used, or the longest interval where none was prespecified (Supplementary Table S3). Because biomarkers measured in the same participants are statistically dependent, the number of results pointing in a given direction is nowhere treated as evidence of consistency, and no directional count is used as a summary.

The protocol prespecified blood, urine or cerebrospinal fluid. Two studies additionally reported LEV-relevant markers in other compartments—salivary superoxide dismutase in one, leukocyte RNA and protein in the other—but both were eligible on blood-based markers, so no study entered on a non-prespecified matrix. Matrix is identified throughout and treated as a source of indirectness in the certainty assessment.

### 2.6. Comparator Streams and Synthesis

Studies were organized into three streams:

- Stream A: within-person change from pretreatment baseline to follow-up on LEV;
- Stream B: LEV-treated participants with epilepsy versus healthy controls;
- Stream C: LEV versus another active antiseizure medication.

Each stream was defined by the randomized trial it approximates, since a risk-of-bias judgement for a non-randomized study is meaningful only against a specified counterfactual; the three target trials are specified in Supplementary Table S8, with the accompanying rationale in Supplementary Note S1. Stream A’s target trial randomizes people with epilepsy to start LEV or not, so an uncontrolled before-after study has no counterfactual arm at all. Stream C’s target trial randomizes newly diagnosed epilepsy to LEV or a specified alternative. Stream B corresponds to no target trial, healthy volunteers being ineligible for the intervention; it is therefore reported as descriptive evidence about treated epilepsy and downgraded for indirectness rather than treated as a confounded treatment effect. The five prespecified subgroup analyses (by condition, duration, biomarker class, design and sub-stream) were all infeasible, so the synthesis is structured by comparator sub-stream and biomarker class.

The prespecified effect measure was the standardized mean difference with 95% confidence intervals, to be pooled in random-effects models; no cell met the conditions for pooling. Pooling and estimation are separate questions, and individual estimates were calculated wherever source reporting permitted: mean differences in original units with 95% confidence intervals, and Hedges’ g, for between-group contrasts. Only one study reported change-score standard deviations, and they could not be reconciled with the summary statistics printed alongside them (Section 3.3); for the remainder, where an exact paired *p* value was reported and the correlation it implies lies within the possible range, we recovered that correlation and derived the interval without assuming one; where it does not, or where only a significance threshold was reported, intervals are shown under assumed correlations of 0.5 and 0.7 and identified as assumption-dependent. Such values are identified as calculated by the review authors, are unadjusted for multiplicity, and characterize precision rather than establish effects.

### 2.7. Risk of Bias

ROBINS-I (2016) was applied to the LEV-relevant result of every study against the target trial specified for its stream [12,13]. Domain-level judgments for all seven domains are in Supplementary Table S4; overall judgments follow the ROBINS-I rule that a study is rated at its most severe domain, which is why they are less differentiated than the domain assessments generating them. Deviations from intended interventions were assessed as adherence, co-medication and post-initiation dose change, no study having involved formal allocation; the judgement for each study is given in Supplementary Table S4. We considered a critical rather than serious judgement for confounding in the uncontrolled before-after studies and did not apply it; the reasoning, and what changes if a reader disagrees, is in Supplementary Note S1. No conclusion depends on that choice.

Separately, every report was passed through a post hoc source-verifiability and reporting-consistency check, because a synthesis can be distorted by a report whose content cannot be reconciled with its own stated methods. Five binary criteria were applied, each verifiable by any reader of the published article: (i) the principal method named in the title or abstract appears in the Methods and generates reported results; (ii) mechanistic statements are attributed to the drug they concern; (iii) numerical results are tabulated rather than figure-only; (iv) the dispersion measure is identified and used consistently; (v) the reference list is free of automated parsing artefacts. Two levels were recorded and are not merged: Level 1, ordinary reporting deficiencies (iii, iv), common here and implying nothing beyond incomplete reporting; Level 2, unresolved discrepancies between what a report states about itself and what it contains (i, ii, v). The check records what is observable in the published text; it is not an investigation and makes no finding about the conduct of any study. Four consequences of a Level 2 result were fixed in advance: the report stays in the descriptive synthesis, cannot be the sole support of a GRADE body, does not contribute to judgments of consistency, and is removed in the sensitivity analysis of Section 3.9. Full operationalization is in Supplementary Note S2.

Risk of bias due to missing results was assessed qualitatively per eligible cell (tabulated, figure-only or unusable result; apparent prespecification; complete reporting of measured biomarkers). Funnel plots and tests for small-study effects were not applicable.

### 2.8. Certainty of Evidence

GRADE was applied separately to bodies of evidence defined by comparator stream and outcome domain [14]. Non-randomized intervention evidence began at low certainty and was further downgraded when warranted for serious risk of bias, inconsistency, indirectness, imprecision, or suspected publication and reporting bias. No upgrading criterion was met. Because most bodies contained one to four small studies and did not provide a common effect estimate, certainty judgments emphasize the range and credibility of possible effects rather than a pooled magnitude.

## 3. Results

### 3.1. Study Selection

**Figure 1:**
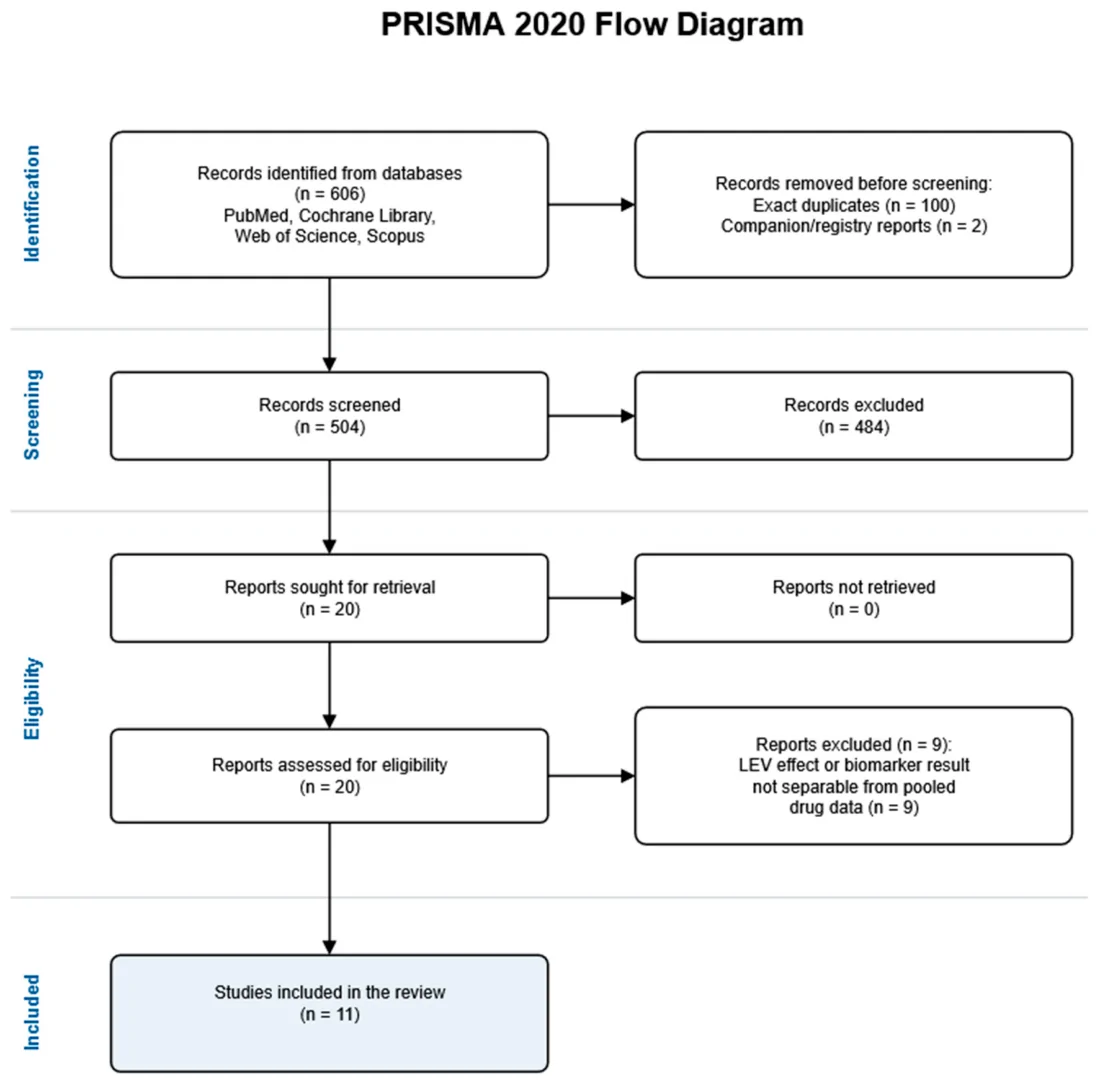
PRISMA 2020 flow diagram for study identification and selection. LEV, levetiracetam.

The searches identified 606 records. After removing 100 exact duplicates and the two linked companion records described in Section 2.4, 504 records underwent title/abstract screening and 484 were excluded. All 20 reports sought for full-text assessment were retrieved. Eleven studies were included; nine were excluded at full text because LEV-specific exposure effects or biomarker results were not separable from pooled drug data (Figure 1), each with its reason in Supplementary Table S6. The database of origin for every included study is given in Supplementary Table S7.

### 3.2. Study Characteristics

The 11 studies were published from 2010 to 2025 in Turkey, Egypt, Iran, India and China (Table 1; full extraction in Supplementary Table S2). All enrolled people had epilepsy; no study of another condition was eligible, and no randomized trial provided an eligible LEV-specific outcome. Five studies contributed within-person data, four compared LEV-treated participants with healthy controls, and two contributed principally active-drug comparisons; two further studies also included an active comparator. Two studies were pediatric.

The 297 participants are those with analysable LEV-relevant biomarker data, not those enrolled: the figure excludes 22 of the 58 LEV-treated adults of Sarangi et al. [25] in whom total antioxidant capacity was not measured, 4 of 34 in Mehvari-Habibabadi et al. [22] and 3 of 15 in Swathi and Aruna [23]. A single total ever exposed to LEV cannot be derived, because Elsayed et al [20]. gave no drug-specific account of attrition. Matrices included serum, plasma, saliva, urine, leukocytes and leukocyte RNA or protein.

The clinical features that would determine whether divergent findings reflect different populations are set out in Table 2 and are largely absent from the sources. Monotherapy status is identifiable in nine studies and exposure duration in eight, but seizure frequency is reported in four studies and in only one of them for the on-treatment period; treatment response is reported in a single study (Swathi and Aruna [23], as the proportion seizure-free at three months); and the interval between the last seizure and biomarker sampling is standardized in two—Ozden et al. [16], which required at least one month of seizure freedom for inclusion, and Wang et al. [24], which required 24 h. Dose is absent in four studies.

### 3.3. Availability of Quantitative Synthesis

**Figure 2:**
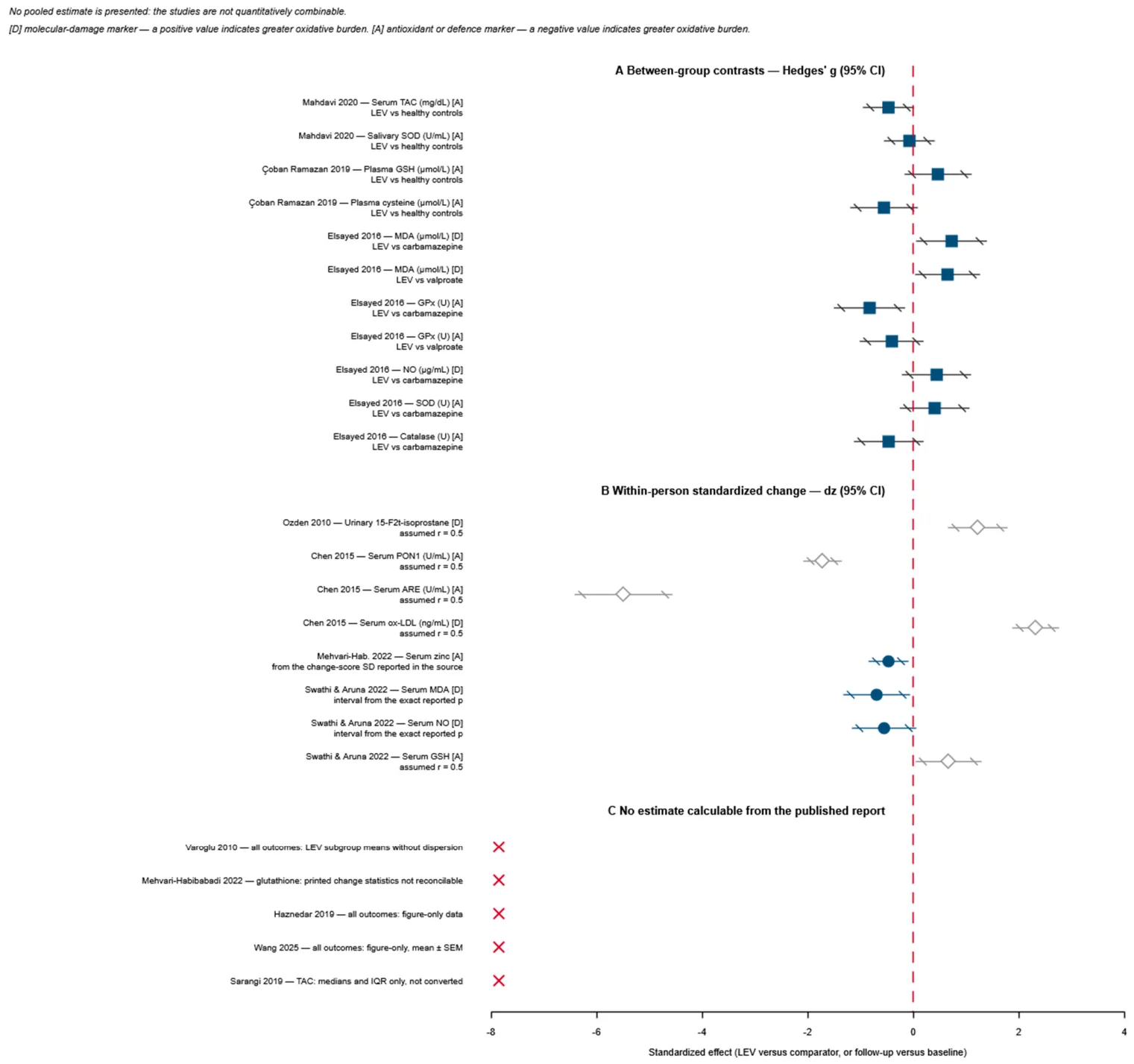
Effect estimates with 95% confidence intervals for every LEV-relevant result from which one could be calculated, and the studies for which none could. No pooled estimate is shown, because the studies are not quantitatively combinable. All estimates were calculated by the review authors from the summary statistics published in the source reports and are unadjusted for multiplicity. Panel (**A**), between-group contrasts (Hedges’ g). Panel (**B**), within-person standardized change (dz); filled markers denote intervals derived without assuming a correlation—either from an exact reported *p* value or, for Mehvari-Habibabadi et al. [22], from the change-score standard deviation printed in the source—and open marker intervals that depend on an assumed within-person correlation of 0.5. Panel (**C**), studies whose reporting permits no estimate. [D] molecular-damage marker; [A] antioxidant or defence marker. The studies shown are Varoglu 2010 [15], Ozden 2010 [16], Chen 2015 [17], Haznedar 2019 [18], Mahdavi 2020 [19], Elsayed 2016 [20], Çoban Ramazan 2019 [21], Mehvari-Habibabadi 2022 [22], Swathi and Aruna 2022 [23], Wang 2025 [24] and Sarangi 2019 [25]. ARE, arylesterase; GPx, glutathione peroxidase; GSH, glutathione; MDA, malondialdehyde; NO, nitric oxide; ox-LDL, oxidized low-density lipoprotein; PON1, paraoxonase-1; SOD, superoxide dismutase; TAC, total antioxidant capacity.

No biomarker-by-comparator cell contained two sufficiently comparable studies with complete variance information, so meta-analysis was not performed. Individual estimates were nonetheless calculated wherever the source permitted, and the pattern of what could not be calculated is itself a principal result (Figure 2). Seven of the 11 studies yielded at least one estimate with a confidence interval; four did not—Varoglu et al. [15] reported subgroup means without subgroup dispersion, Haznedar et al. [18] and Wang et al. [24] reported values only in figures, and Sarangi et al. [25] reported medians with interquartile ranges, which were not converted (Section 2.4). One study, Mehvari-Habibabadi et al. [22], reported change-score standard deviations, but they cannot be reconciled with the statistics printed beside them: for zinc the reported change of −6.68 ± 14.11 is close to, but not identical with, the difference between the reported means (−6.62), and yields a 95% confidence interval of −11.95 to −1.41 from which the printed test statistic, confidence interval and *p* value do not follow; for glutathione the printed change of −66.78 matches neither the difference between the printed means (−32.25) nor the printed test statistic, so no defensible interval can be derived. For the remaining paired studies, no change-score standard deviation was reported. Where an exact paired *p* value allowed the implied within-person correlation to be recovered it was plausible for Swathi and Aruna [23]; for Ozden et al. [16] it fell outside the possible range, so the reported *p* of 0.025 cannot be reproduced by a paired t test on the published summary statistics. Two further discrepancies emerged on recalculation: the serum total antioxidant capacity contrast of Mahdavi et al. [19], reported as *p* = 0.02, yields *p* = 0.05 with an interval reaching no difference, whereas the pairwise LEV-versus-carbamazepine contrasts in Elsayed et al. [20] are stronger than that study’s three-group omnibus *p* suggests. Neither is a correction of the source; both are review-calculated, unadjusted for multiplicity, and reported so readers can see the precision behind the published claims.

### 3.4. Stream A: Within-Person Change After LEV

All five within-person comparisons were rated at serious risk of bias (Table 3). None included a concurrent untreated epilepsy group, so none can distinguish a pharmacological effect of LEV from seizure control, time or regression to the mean, and only one reported the change-score variance a paired estimate requires, in a form that cannot be reconciled with its own summary statistics (Section 3.3). What follows describes what each report published within its own cohort; the intervals in Table 4 should be read before the point estimates. The estimates calculable in this and the other two streams are given in Table 4.

Ozden et al. studied 21 adults with cryptogenic partial epilepsy in an uncontrolled before-after design, excluding 9 of 30 initially considered for a seizure in the preceding month; the analyzed cohort was therefore selected on recent seizure freedom and had no untreated comparator, and its reported *p* value is not reproducible by a paired t test (Section 3.3) [16]. Urinary 15-F2t-isoprostane rose over three months (Table 4). The direction is compatible with increased lipid peroxidation; the magnitude cannot be attributed to LEV.

Chen et al. studied 72 people with refractory epilepsy before and after LEV without reporting the follow-up interval or characterizing prior and concomitant therapy, so neither exposure nor counterfactual is defined [17]. The source abstract contradicts itself on the direction of oxidized LDL; we resolved this in favour of the numerical table and made no silent correction. Paraoxonase-1 and arylesterase fell and oxidized LDL rose (Table 4), but the standardized changes implied by the published standard deviations are extreme for circulating biomarkers, which argues for caution about the reported dispersion rather than for a large effect.

Mehvari-Habibabadi et al. followed 30 of 34 participants for three months in an uncontrolled design [22]. It is the only included study to report change-score standard deviations, and neither of its two review-relevant outcomes is internally coherent. For zinc the printed change of −6.68 ± 14.11 does not exactly match the difference between the printed means (−6.62), and the accompanying test statistic, confidence interval and *p* value follow from neither; the Abstract and Discussion also describe as non-significant a change the table reports at *p* < 0.001. For glutathione the printed change of −66.78 agrees neither with the difference between the printed means (−32.25) nor with the printed test statistic. Zinc is therefore classified as decreased with unresolved statistical uncertainty rather than a confirmed oxidative effect, and glutathione as not interpretable (Table 4).

Swathi and Aruna analyzed 12 of 15 LEV-assigned, newly diagnosed drug-naive adults after three months; selection was non-random, attrition incompletely explained and no untreated group included, so the drug-naive baseline strengthens temporal ordering without supplying a counterfactual [23]. Malondialdehyde and nitric oxide fell and glutathione rose, an internally coherent pattern, but the intervals are wide relative to the changes: the malondialdehyde interval excludes no change only marginally and the nitric oxide interval includes it (Table 4).

Wang et al. evaluated 32 first-medication adults receiving LEV [24]. This is the only included report with a Level 2 result on the check of Section 2.7. The three features producing that result are stated below as observations about the published text, without inference about how the study was conducted: the principal method named in the title, thermal infrared imaging, does not appear in the Methods or Results and generates no reported data; a passage introduced as describing levetiracetam abbreviates it ‘LTG’ and attributes to it the sodium-channel mechanism of lamotrigine; and the reference list contains automated parsing artefacts. The report separately fails the two Level 1 criteria, which concern reporting completeness alone: every biomarker outcome is presented only as a figure-based mean with a standard error, so no estimate could be calculated. The report describes lower Fe^2+^ and malondialdehyde and higher glutathione, GPX4 and SLC7A11 after treatment, without dose, duration or a concurrent untreated group. Peripheral expression changes in GPX4 and SLC7A11 do not demonstrate neuronal ferroptosis or its inhibition. Under Section 2.7 the report remains in the descriptive synthesis, is not the sole support of any certainty rating, contributes nothing to judgments of consistency, and is excluded in Section 3.9.

Within-person evidence does not converge. Cohorts of treated or refractory epilepsy report increased oxidative burden while drug-naive and first-medication cohorts report a broadly protective pattern; the studies differ simultaneously in treatment history, refractoriness, follow-up, biomarker panel and completeness of reporting, and no two report the same biomarker in comparable patients with comparable variance information. This is deliberately not summarized as a count of studies in each direction: results within a cohort are statistically dependent, sample sizes range from 12 to 72, and the calculable intervals in Table 4 overlap almost everywhere.

### 3.5. Stream B: LEV-Treated Epilepsy Versus Healthy Controls

This stream corresponds to no target trial (Section 2.6): every comparison is between people with epilepsy receiving LEV and people without epilepsy, so exposure and disease are inseparable by design. All four comparisons were rated at serious risk of bias, and two report no usable dispersion for the LEV subgroup.

Varoglu et al. reported means for a LEV monotherapy subgroup of eight without subgroup dispersion, so no estimate is calculable; the article states 61 patients while its treatment-group counts sum to 68, and no silent correction was made [15]. Arylesterase was lower and 8-hydroxyguanine higher than in controls; paraoxonase-1 and oxidized LDL were not identified as different.

Haznedar et al. compared 26 children on LEV monotherapy for at least six months with 26 healthy children and 25 on valproate, presenting all means and dispersions only graphically, so no estimate is calculable [18]. Malondialdehyde and 8-hydroxy-2′-deoxyguanosine were higher than in healthy controls, and malondialdehyde also higher than with valproate; the valproate contrast is the more informative because it holds disease status constant.

Mahdavi et al. compared 33 adults on LEV monotherapy for at least six months with 35 healthy controls in whom vitamin C intake also differed, so diet is confounded with exposure alongside disease [19]. Serum total antioxidant capacity was lower with LEV, but the recalculated interval reaches no difference and the recalculated *p* is 0.05 rather than the 0.02 reported (Section 3.3); salivary superoxide dismutase did not differ, with an interval consistent with a moderate difference in either direction (Table 4).

Çoban Ramazan et al. compared 13 LEV-treated participants with 38 healthy controls; glutathione and cysteinylglycine peaks were incompletely separated in some chromatograms, limiting measurement validity independently of design [21]. No aminothiol difference was identified, and the calculated intervals show why: both the glutathione and the cysteine interval span moderately favourable to moderately unfavourable effects (Table 4). This is imprecision, not evidence of no effect.

Healthy-control comparisons tend to show more molecular damage or lower antioxidant capacity in LEV-treated epilepsy. The same direction would be expected if epilepsy or recent seizures raised oxidative burden irrespective of medication, and no design feature of these studies can separate the two.

### 3.6. Stream C: LEV Versus Other Antiseizure Medications

Both comparisons in this stream were rated at serious risk of bias for confounding by indication, and the comparator drug differs between them.

Elsayed et al. followed children with newly diagnosed epilepsy for six months but reported baseline biomarkers only for the pooled cohort, so no LEV-specific before-after effect is calculable; 26 of 110 enrolled children were absent at follow-up without a drug-specific attrition account [20]. The source reports a three-group omnibus comparison and states in its Results that multiple comparison tests between individual drugs were performed, but prints no pairwise *p* values; the pairwise contrasts calculated here show higher malondialdehyde and lower glutathione peroxidase with LEV than with carbamazepine, both with intervals excluding no difference; against valproate the malondialdehyde difference is of the same magnitude and its interval also excludes no difference, whereas the glutathione peroxidase difference is smaller and its interval does not (Table 4). These are review-calculated, unadjusted for multiplicity, from a non-randomized comparison of 14 LEV-treated children with 26 and 44 on other drugs.

Sarangi et al. measured total antioxidant capacity in a consecutively recruited subset of 36 LEV-treated and 38 conventionally treated adults, with a comparator pooling carbamazepine, phenytoin and valproate [25]. Results were reported as medians with interquartile ranges, which were not converted (Section 2.4), so no standardized estimate is available. The source reports no total antioxidant capacity value for the LEV group or the conventional group as a whole: the only LEV-versus-conventional *p* value it prints (0.610) compares the two groups within the subgroup positive for psychiatric and behavioural adverse effects (23 versus 13 participants). This review therefore records no detected difference in that subgroup, which supports neither a group-level difference nor equivalence.

Two further studies contributed active-comparator contrasts within other designs: Haznedar et al. [18] reported higher malondialdehyde with LEV than with valproate, and Wang et al. [24] included active-drug groups reported only graphically. Across the stream the comparator drug differs in every contrast, no two studies compare the same pair, and the evidence cannot rank LEV against any alternative medication.

### 3.7. Risk of Bias

Every LEV-relevant comparison was rated serious overall (Table 3), but the domain-level judgments producing those ratings are not uniform and are given in full in Supplementary Table S4. Confounding was serious in all 11 studies for different reasons by stream: structural inseparability of disease and exposure in Stream B, absence of any counterfactual in Stream A, confounding by indication in Stream C. Outcome measurement was serious in one study only, and reporting of results in six. Section 2.7 states why no study was rated critical and what follows if a reader disagrees.

Study-specific drivers are listed in Table 3 and detailed in Supplementary Table S4. Selective reporting could not be excluded in ten of the eleven studies, which assessed multiple biomarkers without a prespecified primary outcome or a public analysis plan. The exception is Swathi and Aruna [23], whose linked registry record (Section 2.4) provides a prespecified outcome set; that record was checked against the publication and no measured biomarker was unreported.

### 3.8. Certainty of Evidence

Certainty was very low for every outcome-domain/comparator body (Table 5; full profile in Supplementary Table S5). Within-person bodies were downgraded for serious risk of bias, non-convergent directions across small cohorts and imprecision. Healthy-control bodies were downgraded principally for indirectness, the contrast being one no trial could randomize. Active-comparator bodies were sparse, non-randomized and based on comparator drugs differing between studies. The ferroptosis-related body rests on a single report with a Level 2 verifiability result and is retained only under the constraints of Section 2.7. No body supported a precise effect magnitude or met any criterion for upgrading.

### 3.9. Sensitivity Analysis Excluding the Report with a Verifiability Concern

Under the rules fixed in Section 2.7, a post hoc sensitivity analysis excluded the single report with a Level 2 verifiability result (Wang et al. [24]) from every synthesis. Ten studies and 265 participants with LEV-relevant biomarker data remained. Within-person evidence continued not to converge: the remaining cohorts still divided between increased oxidative burden in treated or refractory epilepsy and a broadly protective pattern in the single drug-naive cohort, with one cohort not interpretable. Healthy-control comparisons were unaffected, that report having contributed none. Active-comparator evidence remained sparse and heterogeneous in the comparator drug. No certainty rating changed: every remaining body stayed at very low certainty, and because no estimate could be calculated from that report, no interval in Table 4 changes. The one substantive consequence is that the ferroptosis-related body of evidence disappears entirely: in the sensitivity analysis, no human evidence on ferroptosis-related markers during LEV treatment remains. Every conclusion of this review is therefore robust to the exclusion of this report, and the ferroptosis findings should be read as resting on it alone.

## 4. Discussion

### 4.1. Principal Findings

The principal result is not a direction of effect but a characterization of why no direction can be established. Four of eleven studies report outcomes in a form from which no estimate can be derived. Of those that do, the intervals are wide, or depend on an assumed within-person correlation, or arise from uncontrolled comparisons in which no interval can be attributed to the drug; no effect magnitude is replicated for any biomarker in any comparator stream. No two studies report the same biomarker, in the same matrix, under the same estimand, with the variance information a comparison requires. The most direct evidence—within-person change—divides along a line tracking treatment history and cohort type rather than any measured property of the drug, and the healthy-control contrast, which supplies the most consistent findings, answers a question no trial could ask.

The evidence is therefore not summarized here by counting studies in each direction. A single study may report simultaneous changes in damage products, enzymes and substrates that lack equivalent biological meaning and are measured in the same people; a count treats these as independent votes and weights a study of 12 participants equally with one of 72. No effect magnitude is replicated for any biomarker in any stream.

### 4.2. Separating Medication Effects from Epilepsy and Seizure Control

Seizures acutely increase mitochondrial oxidant production, lipid peroxidation and nucleic-acid damage [2,3], and sampling time relative to the last seizure was almost never standardized. The resulting bias runs in both directions: a treated-versus-healthy difference may be wrongly attributed to LEV when it reflects epilepsy or recent seizures, while an improvement after LEV may reflect fewer seizures rather than antioxidant action.

**Figure 3:**
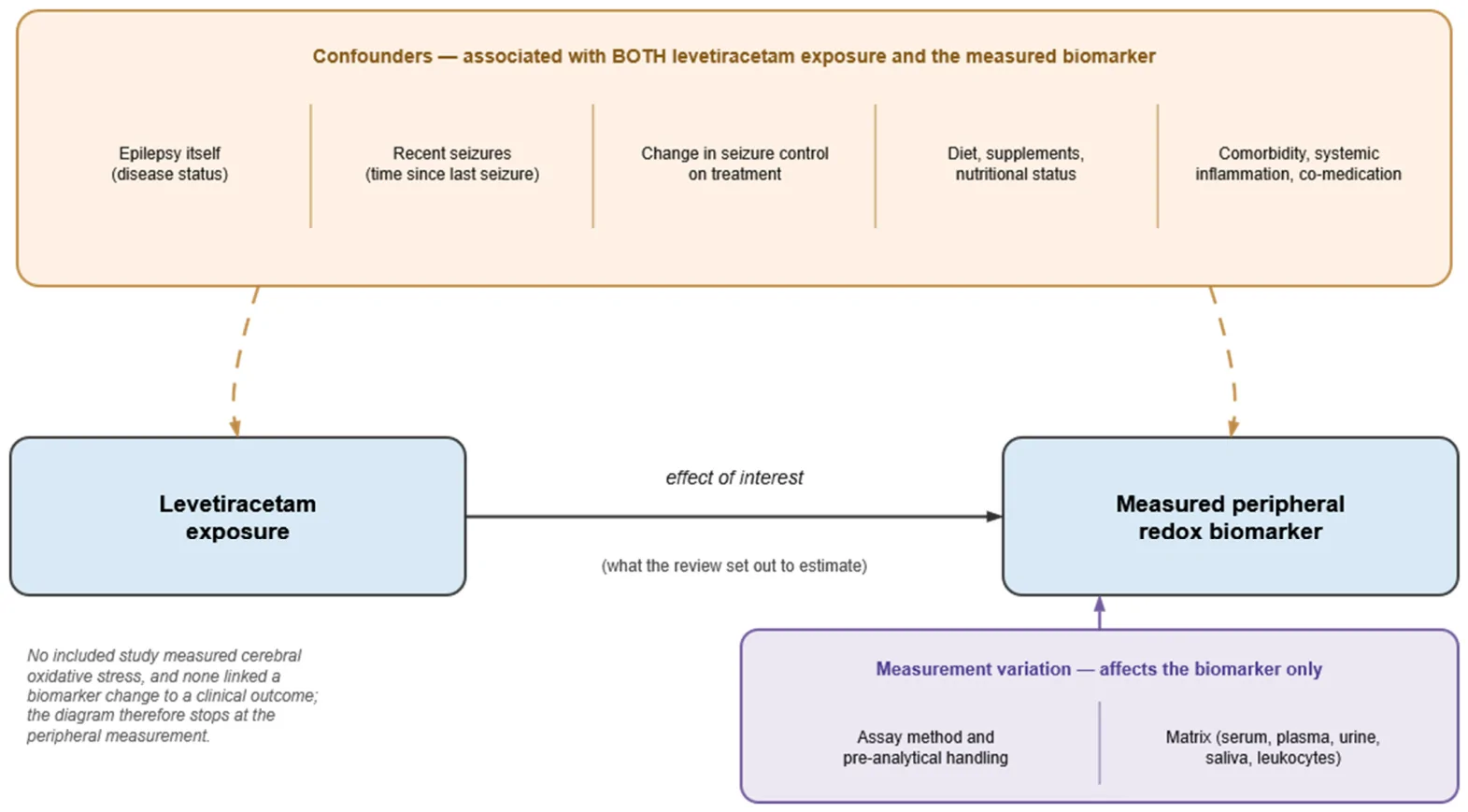
Principal sources of confounding and measurement variation separating levetiracetam exposure from the measured peripheral redox biomarker. Confounders ((**upper**) panel) are associated with both exposure and outcome and cannot be removed by any of the included designs; measurement factors ((**lower**) panel) affect the biomarker alone. The diagram stops at the peripheral measurement because no included study measured cerebral oxidative stress or linked a biomarker change to a clinical outcome (Section 4.4).

The contrast between refractory and drug-naive cohorts is notable but not causal proof, and Table 2 and Figure 3 show why it cannot be resolved from the published literature: treatment response is reported in one study, on-treatment seizure frequency in one, and a standardized interval between the last seizure and sampling in two. The variables that would explain the divergence are precisely those the literature does not report.

### 4.3. Interpretation of Biomarker Domains

Damage products and antioxidant defences answer different questions. F2-isoprostanes and oxidized DNA products index accumulated molecular oxidation more directly than any single enzyme activity, whereas malondialdehyde is sensitive to pre-analytical handling and assay specificity, particularly with TBARS-based methods [8,9]. Total antioxidant capacity is a composite affected by urate, albumin and diet. Antioxidant enzymes may fall through depletion or inhibition but may equally rise as adaptive responses, so higher activity is not invariably beneficial and lower activity is not by itself evidence of drug toxicity. Circulating glutathione does not reflect brain glutathione, zinc is determined by diet, inflammation and protein binding, and Fe^2+^, GPX4 and SLC7A11 do not demonstrate ferroptotic neuronal death when measured peripherally.

### 4.4. From Biomarker Change to Clinical Meaning

A recurring difficulty in this literature is that a change in a circulating biomarker is discussed as though it were a clinical effect. Five distinct claims are involved and are not interchangeable: (i) a peripheral biochemical change; (ii) a change in cerebral oxidative stress; (iii) neuroprotection or neurotoxicity; (iv) an effect on antiseizure efficacy; and (v) an effect on clinical safety. Each step requires evidence the previous one does not supply.

The included literature occupies the first rung only. All 11 studies measured peripheral biochemistry. None measured cerebral oxidative stress, in cerebrospinal fluid or by imaging. None assessed a neuroprotective or neurotoxic outcome. None related biomarker change to seizure control, and none related it to an adverse-effect profile or any other clinical endpoint. No included study established a biomarker threshold predicting benefit or harm.

Two consequences follow. A change in malondialdehyde, total antioxidant capacity or GPX4 reported in these studies is not itself a benefit or a harm; describing a fall in malondialdehyde as protective, or a rise in isoprostanes as toxicity, imports a claim about rungs (ii) to (v) that no included study supports. And independently of whether LEV has any redox effect, measuring oxidative biomarkers to guide levetiracetam prescribing or monitoring cannot be recommended, because the evidence needed to interpret such a measurement clinically does not exist. This, rather than the direction of any individual finding, is the conclusion with practical consequences for clinicians.

### 4.5. Relationship to Experimental Evidence

Experimental work is itself divided. Rodent models of temporal lobe epilepsy describe modulation of mitochondrial dysfunction, inflammatory signalling and antioxidant enzyme activity [5,6], whereas LEV given to non-epileptic animals increased hippocampal DNA oxidation and oxidized glutathione [7]. This demonstrates context dependence rather than resolving the human question: experimental doses, brain-tissue measurement, induced seizure models and treatment timing differ fundamentally from circulating biomarkers during routine treatment.

Two further human studies bear on the context without providing a LEV-specific estimate: Ersan et al., reporting no detected difference among LEV, valproate and carbamazepine but publishing no LEV-stratified values [26], and Martinc et al., which pooled LEV with pregabalin and topiramate [27]. Ersan et al. was assessed at full text and excluded for the absence of a separable LEV result (Supplementary Table S6); Martinc et al. was not eligible for full-text assessment for the same reason and is cited only as context. The remaining eight reports excluded at full text for the same reason, each with its individual justification, are listed in Supplementary Table S6 [28,29,30,31,32,33,34,35].

### 4.6. Strengths and Limitations of the Evidence Synthesis

The principal strengths are the separation of three comparator estimands against explicitly specified target trials, the use of individual biomarker results rather than study-level votes, and the calculation of effect estimates with confidence intervals wherever source reporting allowed, which converts an impression of inconsistency into a measurable statement about precision. Reconciliation against full texts identified discrepancies that would otherwise alter interpretation: the Varoglu denominator [15], the Chen oxidized-LDL wording conflict [17], the absence of LEV-specific baseline data in Elsayed et al. [20], the Sarangi biomarker subset [25], the Mehvari zinc inconsistency [22], and the non-reproducibility of two published *p* values (Section 3.3).

The review is limited chiefly by the primary evidence: all included studies were non-randomized and mostly small, key confounders were rarely controlled, dose or duration was often absent, sampling relative to seizures was almost never standardized, and several reports were figure-only, incomplete or internally inconsistent.

Coverage was also incomplete, and we do not minimize it. Embase was not searched because no access was available (Section 2.3), and Supplementary Table S7 argues that this matters: three of eleven included studies were retrieved by a single database each, and no database retrieved more than nine, so in a literature indexed this unevenly an unsearched database may well contain eligible reports. Trial registries and grey literature were not systematically searched. The English-language restriction was prespecified but remains a limitation: it may under-represent studies from countries that contributed several included reports, and the direction of any resulting bias is unpredictable rather than conservative. Selective-outcome and publication bias could not be assessed statistically and remain plausible. These limitations reinforce rather than qualify the central conclusion: an evidence base that cannot be shown to be complete, and whose retrieved members cannot answer the question, does not become more informative by being enlarged with reports of the same design.

### 4.7. Research Recommendations

The highest-value future design would enrol drug-naive people with newly diagnosed epilepsy and compare LEV with an active treatment under random allocation; where randomization is not feasible, prospective target-trial emulation with prespecified confounder adjustment is required. Studies should:

- prespecify one or more primary redox outcomes and a limited mechanistically coherent panel;
- standardize fasting status, collection time, processing, storage, and time since the last seizure;
- report baseline seizure burden and change in seizure frequency;
- document LEV dose, adherence, co-medication, supplements, diet, smoking, BMI, and inflammatory or metabolic disease;
- measure LEV plasma concentration only where an exposure-biomarker relationship is a stated objective, since concentration does not consistently track efficacy or adverse effects in routine practice;
- use validated assays and distinguish plasma, serum, urine, saliva, blood-cell, and brain-relevant measures;
- report exact estimates, dispersion, paired change-score variance, confidence intervals, attrition, and all prespecified outcomes;
- include a concurrent epilepsy comparator to separate treatment effects from the disease and from seizure control;
- test whether biomarker changes correlate with clinically meaningful outcomes rather than treating surrogate changes as clinical benefit or harm;
- prespecify and power for candidate effect modifiers rather than testing them post hoc—baseline oxidative status, baseline and on-treatment seizure burden, comorbid metabolic disease and, exploratorily, pharmacogenetic profile. No included study tested effect modification, so this review provides no evidence for or against such subgroups and can only define what detecting them would require; individualized redox-informed treatment selection would become defensible only if a reproducible subgroup difference were demonstrated and biomarker change were linked to a patient-relevant outcome.

## 5. Conclusions

No body of human evidence identified by this review is of sufficient validity to support or to refute a redox effect of levetiracetam. This is a statement about the evidence, not about the drug: the question has not been answered, not answered in the negative. Four of eleven studies report outcomes in a form from which no estimate can be derived; the intervals that can be calculated are wide, inconsistent in direction, or derived from designs that cannot attribute a change to the drug; certainty is very low in every comparator stream and biomarker domain; and the conclusion is unchanged when the one report with a verifiability concern is removed, ferroptosis-related evidence resting on that report alone. The appropriate clinical conclusion is therefore not that LEV is pro-oxidant, antioxidant or redox-neutral, but that its human redox effects remain unresolved, and that oxidative biomarkers should not be used to guide levetiracetam prescribing or monitoring.

## Figures and Tables

**Table 1:** Characteristics of included studies.

Study	Design and Primary Comparator	Population	LEV *n* with Biomarker Data	Exposure	Matrix and Markers	Main Finding	Main Limitation
Varoglu 2010 [15]	Cross-sectional; LEV vs. healthy controls	Adults with treated epilepsy	8	Dose and LEV-specific duration not reported	Serum PON1, ARE, ox-LDL; leukocyte DNA 8-OHG	Lower ARE and higher 8-OHG vs. healthy controls	LEV subgroup *n* = 8 without dispersion; patient count 61 vs. 68 discrepancy
Ozden 2010 [16]	Prospective single-arm pre-post	Adults with cryptogenic partial epilepsy	21	1000–3000 mg/day; 3 months	Urinary 15-F2t-isoprostane	Urinary 15-F2t-isoprostane increased after 3 months	No untreated comparator; cohort selected on recent seizure freedom
Chen 2015 [17]	Prospective single-arm pre-post	Adolescents/adults with refractory epilepsy	72	20–40 mg/kg/day; duration unreported	Serum PON1, ARE, ox-LDL	PON1 and ARE decreased; ox-LDL increased	Follow-up interval and co-treatment not reported; abstract self-contradictory
Haznedar 2019 [18]	Cross-sectional; LEV vs. healthy and VPA groups	Children with generalized epilepsy	26	20–60 mg/kg/day; at least 6 months	Serum MDA, 8-OHdG	MDA and 8-OHdG higher than healthy controls; MDA higher than VPA	All values figure-only; healthy-control contrast confounded by epilepsy
Mahdavi 2020 [19]	Case–control; LEV vs. healthy controls	Adults with focal/generalized epilepsy	33	Mean 992 mg/day; at least 6 months	Serum TAC; salivary SOD	Serum TAC lower with LEV; salivary SOD unchanged	Cross-sectional; vitamin C intake differed; recalculated *p* = 0.05
Elsayed 2016 [20]	Prospective cohort; post-treatment active-drug comparison	Children with newly diagnosed epilepsy	14	Dose unreported; 6 months	MDA, NO, GPx, SOD, catalase	Lowest GPx across drug groups with LEV; MDA numerically highest	No LEV-specific baseline; attrition not accounted for by drug
Çoban Ramazan 2019 [21]	Cross-sectional; LEV vs. healthy controls	Adolescents/adults with idiopathic epilepsy	13	Dose unreported; mean duration 7.25 ± 5.47 y	Plasma GSH and aminothiols	No aminothiol difference identified vs. healthy controls	Chromatographic peak non-separation; intervals wide in both directions
Mehvari-Habibabadi 2022 [22]	Prospective single-arm pre-post	Adolescents/adults with epilepsy	30	1000–3000 mg/day; 3 months	Serum GSH, zinc	GSH not interpretable; zinc decreased	Zinc and glutathione statistics internally inconsistent in the source
Swathi and Aruna 2022 [23]	Open-label non-randomized pre-post	Drug-naive adults with newly diagnosed epilepsy	12	500 mg twice daily; 3 months	Serum MDA, NO, GSH	MDA and NO decreased; GSH increased	*n* = 12; no untreated control; attrition incompletely explained
Wang 2025 [24]	Prospective pre-post with healthy and active-drug groups	First-medication adults with epilepsy	32	Dose and duration unreported	Fe^2+^, MDA, GSH, GPX4, SLC7A11	Broadly protective peripheral ferroptosis-related pattern	Figure-only data; Level 2 result on the check of Section 2.7
Sarangi 2019 [25]	Cross-sectional; LEV vs. conventional ASMs	Adults on monotherapy	36	1000–3000 mg/day; at least 6 months	Serum TAC	No difference detected within the subgroup positive for psychiatric/behavioural adverse effects	Source prints no TAC value for either treatment group as a whole; medians and IQRs only; selected biomarker subset

ARE, arylesterase; ASM, antiseizure medication; GPx, glutathione peroxidase; GPX4, glutathione peroxidase 4; GSH, glutathione; LEV, levetiracetam; MDA, malondialdehyde; NO, nitric oxide; 8-OHdG, 8-hydroxy-2′-deoxyguanosine; 8-OHG, 8-hydroxyguanine; ox-LDL, oxidized low-density lipoprotein; PON1, paraoxonase-1; SLC7A11, solute carrier family 7 member 11; SOD, superoxide dismutase; TAC, total antioxidant capacity; VPA, valproate. “LEV *n* with biomarker data” is the number of LEV-treated participants with analysable biomarker data and sums to 297. The final two columns separate the observed finding from the principal limitation on its interpretation.

**Table 2:** Clinical and treatment context of the included studies.

Study	Epilepsy Stage	Age Group	Mono- vs. Polytherapy	Seizure Frequency Reported	Treatment Response Reported	Standardized Time Since Last Seizure	LEV Dose	LEV Duration
Varoglu 2010 [15]	Established, treated	Adults	Monotherapy subgroup (*n* = 8)	Yes (cohort range, 0–15/month)	No	No	NR	NR (LEV-specific)
Ozden 2010 [16]	Cryptogenic partial, on LEV	Adults	NR	No	No	Yes (≥1 month, inclusion criterion)	1000–3000 mg/day	3 months
Chen 2015 [17]	Refractory	Adolescents/adults	Unclear; co-treatment not described	No	No	No	20–40 mg/kg/day	NR
Elsayed 2016 [20]	Newly diagnosed	Children	Monotherapy (by drug group)	No	No	No	NR	6 months
Haznedar 2019 [18]	Generalized, established	Children	Monotherapy (polytherapy excluded)	No	No	No	20–60 mg/kg/day	≥6 months
Mahdavi 2020 [19]	Focal or generalized, established	Adults	Monotherapy	No	No	No	Mean 992 mg/day	≥6 months; mean 2.24 y
Çoban Ramazan 2019 [21]	Idiopathic, established	Adolescents/adults	Monotherapy	No	No	No	NR	Mean 7.25 y
Mehvari-Habibabadi 2022 [22]	Established	Adolescents/adults	Monotherapy	Yes (baseline categories)	No	No	1000–3000 mg/day	3 months
Swathi and Aruna 2022 [23]	Newly diagnosed, drug-naive	Adults	Monotherapy	Yes (baseline)	Yes (7/12 seizure-free at 3 months)	No	500 mg twice daily	3 months
Wang 2025 [24]	First medication	Adults	Monotherapy	No	No	Yes (≥24 h, interictal sampling)	NR	NR
Sarangi 2019 [25]	Established	Adults	Monotherapy	Yes (per person, last 6 months, by group)	No	No	1000–3000 mg/day	≥6 months
Number of studies reporting	11/11	11/11	9/11	4/11 (1 on treatment)	1/11	2/11	7/11	8/11

LEV, levetiracetam; NR, not reported. Epilepsy stage and age group are reported as defined by the original authors; children, <12 years; adolescents/adults, ≥12 years. “Seizure frequency reported” denotes a quantitative account of seizures at baseline or on treatment; "treatment response reported", any reported change in seizure frequency attributable to treatment; “standardized time since last seizure”, a protocol-defined interval between the last seizure and biomarker sampling. Cells marked "unclear" indicate that the item could not be determined from the published report. The final row gives the number of studies reporting each item; “on treatment” in that row means seizure frequency reported for the treated period and identifiable for the LEV group, whereas a whole-cohort range spanning several drugs is counted as reported but not as on treatment.

**Table 3:** Principal risk-of-bias concerns for each LEV-relevant comparison.

Study	Confounding/Selection	Exposure and Deviations	Missing Data/Measurement/Reporting	Overall Interpretation
Varoglu 2010 [15]	Epilepsy vs. health; group-count inconsistency	Dose/duration absent	Subgroup dispersion absent	Serious
Ozden 2010 [16]	No concurrent control; recent-seizure selection	Adherence incompletely described	9/30 excluded before analysis	Serious
Chen 2015 [17]	Refractory uncontrolled cohort	Duration/co-medication unclear	Abstract/table wording conflict	Serious
Haznedar 2019 [18]	Epilepsy vs. health	Monotherapy reasonably defined	Figure-only key outcomes	Serious
Mahdavi 2020 [19]	Epilepsy and vitamin C intake differ	Monotherapy defined	Cross-sectional surrogate outcomes	Serious
Elsayed 2016 [20]	Non-random drug selection	Dose absent	Attrition; no LEV baseline	Serious
Çoban Ramazan 2019 [21]	Epilepsy vs. health	Dose absent	Incomplete chromatographic separation	Serious
Mehvari-Habibabadi 2022 [22]	No concurrent control	Exposure reasonably defined	Attrition and irreconcilable zinc statistics	Serious
Swathi and Aruna 2022 [23]	Non-random selection; no untreated control	Fixed dose	3/15 LEV participants not analyzed	Serious
Wang 2025 [24]	No untreated epilepsy comparator	Dose/duration absent	Figure-only outcomes; selective-reporting concern	Serious
Sarangi 2019 [25]	Confounding by indication; heterogeneous comparator	Monotherapy defined	Selected biomarker subset	Serious

LEV, levetiracetam. Judgments are those of the review authors and follow the ROBINS-I domains detailed in Supplementary Table S4. Domain-level judgments are not uniform; the overall judgement is set by the most severe domain, per ROBINS-I. Section 2.7 states why no study was rated at critical risk.

**Table 4:** Effect estimates with 95% confidence intervals, calculated by the review authors.

Study	Stream	Outcome	Comparison	Effect Estimate (95% CI), Calculated by the Review Authors	Basis
Mahdavi 2020 [19]	B	Serum TAC (mg/dL)	LEV vs. healthy controls	MD −3.2 (−6.400 to 0.000); Hedges g −0.474 (−0.96 to 0.01)	Means and SDs; source *p* 0.02
Mahdavi 2020 [19]	B	Salivary SOD (U/mL)	LEV vs. healthy controls	MD −0.7 (−4.913 to 3.513); Hedges g −0.079 (−0.56 to 0.40)	Means and SDs; source *p* 0.75
Çoban Ramazan 2019 [21]	B	Plasma GSH (µmol/L)	LEV vs. healthy controls	MD 1.12 (−0.220 to 2.460); Hedges g 0.463 (−0.17 to 1.10)	Means and SDs; source *p* NS
Çoban Ramazan 2019 [21]	B	Plasma cysteine (µmol/L)	LEV vs. healthy controls	MD −24.58 (−51.955 to 2.795); Hedges g −0.563 (−1.20 to 0.08)	Means and SDs; source *p* NS
Elsayed 2016 [20]	C	MDA (µmol/L)	LEV vs. carbamazepine	MD 1.9 (0.023 to 3.777); Hedges g 0.721 (0.05 to 1.39)	Means and SDs; source *p* 0.078 across 3 groups
Elsayed 2016 [20]	C	MDA (µmol/L)	LEV vs. valproate	MD 1.9 (0.054 to 3.746); Hedges g 0.646 (0.03 to 1.26)	Means and SDs; source *p* 0.078 across 3 groups
Elsayed 2016 [20]	C	GPx (U)	LEV vs. carbamazepine	MD −11.8 (−19.387 to −4.213); Hedges g −0.832 (−1.51 to −0.16)	Means and SDs; source *p* 0.046 across 3 groups
Elsayed 2016 [20]	C	GPx (U)	LEV vs. valproate	MD −5.7 (−11.711 to 0.311); Hedges g −0.413 (−1.02 to 0.19)	Means and SDs; source *p* 0.046 across 3 groups
Elsayed 2016 [20]	C	NO (µg/mL)	LEV vs. carbamazepine	MD 0.3 (−0.207 to 0.807); Hedges g 0.436 (−0.22 to 1.09)	Means and SDs; source *p* 0.415 across 3 groups
Elsayed 2016 [20]	C	SOD (U)	LEV vs. carbamazepine	MD 0.3 (−0.225 to 0.825); Hedges g 0.4 (−0.26 to 1.06)	Means and SDs; source *p* 0.677 across 3 groups
Elsayed 2016 [20]	C	Catalase (U)	LEV vs. carbamazepine	MD −0.5 (−1.353 to 0.353); Hedges g −0.469 (−1.13 to 0.19)	Means and SDs; source *p* 0.325 across 3 groups
Ozden 2010 [16]	A	Urinary 15-F2t-isoprostane	Baseline vs. follow-up on LEV	Mean change 684 (428.483 to 939.517 at r = 0.5; 479.185 to 888.815 at r = 0.7)	Reported *p* (0.025) not reproducible by a paired t test (implied r = −1.912); intervals assumption-dependent
Chen 2015 [17]	A	Serum PON1 (U/mL)	Baseline vs. follow-up on LEV	Mean change −27.5 (−31.242 to −23.758 at r = 0.5; −30.764 to −24.236 at r = 0.7)	Change-score SD not reported; assumption-dependent. Source *p* <0.05
Chen 2015 [17]	A	Serum ARE (U/mL)	Baseline vs. follow-up on LEV	Mean change −27.09 (−28.248 to −25.932 at r = 0.5; −27.992 to −26.188 at r = 0.7)	Change-score SD not reported; assumption-dependent. Source *p* <0.05
Chen 2015 [17]	A	Serum ox-LDL (ng/mL)	Baseline vs. follow-up on LEV	Mean change 34.6 (31.088 to 38.112 at r = 0.5; 31.841 to 37.359 at r = 0.7)	Change-score SD not reported; assumption-dependent. Source *p* <0.05
Mehvari-Habibabadi 2022 [22]	A	Serum GSH	Baseline vs. follow-up on LEV	Not interpretable	Source prints a change of −66.78 ± 166.72, which matches neither the difference between its own means (−32.25) nor its printed t (1.92); no defensible interval can be derived
Mehvari-Habibabadi 2022 [22]	A	Serum zinc	Baseline vs. follow-up on LEV	Mean change −6.68 (95% CI −11.95 to −1.41)	Derived from the change-score SD printed in the source (14.11), the only one reported by any included study; the source’s own printed t, CI and *p* are not compatible with it
Swathi and Aruna 2022 [23]	A	Serum MDA	Baseline vs. follow-up on LEV	Mean change −0.49 (−0.935 to −0.045); dz −0.7	Interval derived from the exact source *p* (0.0338); implied within-person r = 0.903
Swathi and Aruna 2022 [23]	A	Serum NO	Baseline vs. follow-up on LEV	Mean change −6.87 (−14.684 to 0.944); dz −0.559	Interval derived from the exact source *p* (0.0791); implied within-person r = 0.523
Swathi and Aruna 2022 [23]	A	Serum GSH	Baseline vs. follow-up on LEV	Mean change 75.44 (3.004 to 147.876 at r = 0.5; 18.960 to 131.920 at r = 0.7)	Change-score SD not reported; assumption-dependent. Source *p* <0.0001
Varoglu 2010 [15]	—	All outcomes	—	Not calculable	LEV subgroup means reported without subgroup dispersion
Haznedar 2019 [18]	—	All outcomes	—	Not calculable	All means and dispersions presented only in figures
Wang 2025 [24]	—	All outcomes	—	Not calculable	All outcomes presented only as figure-based mean ± SEM
Sarangi 2019 [25]	—	All outcomes	—	Not calculable	Medians with interquartile ranges only; not converted (Section 2.4)

CI, confidence interval; MD, mean difference; NO, nitric oxide; SD, standard deviation; SEM, standard error of the mean. All estimates in this table were calculated by the review authors from the summary statistics published in the source reports; none was reported by the source. They are unadjusted for multiplicity and are presented to characterize precision, not to establish effects. Between-group estimates use Welch’s method for the mean difference and Hedges’ g for the standardized difference. For within-person comparisons only one source (Mehvari-Habibabadi et al. [22]) reported change-score standard deviations, and the statistics printed beside them are not mutually coherent (Section 3.3). For the remainder, where an exact paired p value was reported and the correlation it implies fell within the possible range, that correlation was recovered and the interval derived without assuming one; where it did not, or where only a significance threshold was reported, intervals are given under assumed correlations of 0.5 and 0.7 and are assumption-dependent. Studies for which no estimate could be calculated are listed explicitly at the foot of the table with the reason.

**Table 5:** GRADE summary by comparator and biomarker domain.

Comparator Stream and Outcome Domain	Studies (Sensitivity Analysis)	Pattern Across Studies	Main Certainty Limitations	Certainty
Within-person: oxidative molecular damage	4 (3)	Directions do not converge; no two studies report the same marker with comparable variance information; the intervals that exclude no change are assumption-dependent or come from uncontrolled before-after comparisons	Serious risk of bias, inconsistency, imprecision, incomplete reporting	Very low
Within-person: antioxidant defence and redox substrates	4 (3)	Decreases, increases and indeterminate results across four small cohorts; results within cohorts are statistically dependent	Serious risk of bias, inconsistency, indirect surrogate measures, imprecision	Very low
Healthy controls: molecular damage	2 (2)	Higher damage markers in LEV-treated epilepsy in both studies; neither yields a calculable estimate	Structural indirectness (no corresponding target trial), small samples, incomplete reporting	Very low
Healthy controls: antioxidant capacity and aminothiols	3 (3)	Lower TAC in one study, borderline on recalculation; aminothiols indeterminate with wide intervals	Indirectness, diet confounding, measurement limitations, imprecision	Very low
Active comparators: antioxidant and damage markers	4 (3)	Comparator drug differs in every contrast; no two studies compare the same pair	Confounding by indication, heterogeneous comparators, figure-only data, sparse evidence	Very low
Within-person: ferroptosis-related peripheral markers	1 (0)	Single report, with a Level 2 verifiability result; body disappears entirely in the sensitivity analysis	Single study, peripheral surrogates, dose and duration absent, figure-only data	Very low

LEV, levetiracetam; TAC, total antioxidant capacity. The full evidence profile, with domain-level downgrading judgments, is given in Supplementary Table S5.

## Data Availability

All data generated during this review are contained within the article and its Supplementary Materials or available upon reasonable request. The protocol is available on OSF at https://doi.org/10.17605/OSF.IO/HXA4U, accessed on 10 September 2026. Further inquiries can be directed to the corresponding author.

## Supplementary Materials

The following supporting information can be downloaded at: https://www.mdpi.com/article/10.3390/ijms27188410/s1.

## Abbreviations

The following abbreviations are used in this manuscript:

8-OHdG	8-hydroxy-2′-deoxyguanosine
8-OHG	8-hydroxyguanine
ARE	arylesterase
ASM	antiseizure medication
BMI	Body mass index
CI	confidence interval
DNA	Deoxyribonucleic acid
GPx	glutathione peroxidase
GPX4	glutathione peroxidase 4
GRADE	Grading of Recommendations, Assessment, Development and Evaluations
GSH	glutathione (reduced form)
LEV	levetiracetam
MDA	malondialdehyde
NO	nitric oxide
OSF	Open Science Framework
ox-LDL	oxidized low-density lipoprotein
PON1	paraoxonase-1
PRISMA	Preferred Reporting Items for Systematic Reviews and Meta-Analyses
PROSPERO	International Prospective Register of Systematic Reviews
RNA	Ribonucleic acid
ROBINS-I	Risk Of Bias In Non-randomized Studies of Interventions
SD	standard deviation
SEM	standard error of the mean
SLC7A11	solute carrier family 7 member 11
SOD	superoxide dismutase
SV2A	synaptic vesicle protein 2A
SWiM	Synthesis Without Meta-analysis
TAC	total antioxidant capacity
TBARS	thiobarbituric acid reactive substances
VPA	valproate
MD	mean difference
NR	not reported
ICTRP	International Clinical Trials Registry Platform
