# Supplementary material for: Levetiracetam and Peripheral Oxidative Stress Biomarkers in Epilepsy: A Systematic Review of Human Evidence
Source: Int J Mol Sci. 2026 Sep 21;27(18):8410. doi: 10.3390/ijms27188410 (PMC13607205; doi:10.3390/ijms27188410)
Supplement: Supplementary file 1 [file ijms-27-08410-s001.zip › Supplementary_Material_1_R1.pdf]

Supplementary Material 1

PRISMA 2020 Checklist

Manuscript: Levetiracetam and Peripheral Oxidative-Stress Biomarkers in Epilepsy: A Systematic Review of Human Evidence. International Journal of Molecular Sciences, manuscript ID ijms-4495780.

Registration: PROSPERO CRD420261448016 (https://www.crd.york.ac.uk/PROSPERO/view/CRD420261448016). Protocol: Open Science Framework, https://doi.org/10.17605/OSF.IO/HXA4U.

Table A. PRISMA 2020 checklist (main report).

Section and Topic	Item #	Checklist item	Location where item is reported
**TITLE**			
Title	1	Identify the report as a systematic review.	Title ("… A Systematic Review of Human Evidence"); article-type line on the first page ("Systematic review").
**ABSTRACT**			
Abstract	2	See the PRISMA 2020 for Abstracts checklist.	Abstract. Item-by-item locations are given in Table B below.
**INTRODUCTION**			
Rationale	3	Describe the rationale for the review in the context of existing knowledge.	Section 1, paragraphs 1–3 (oxidative stress in epilepsy; levetiracetam pharmacology and preclinical redox findings; non-interchangeable biomarkers; the three distinct estimands).
Objectives	4	Provide an explicit statement of the objective(s) or question(s) the review addresses.	Section 1, final paragraph: three numbered objectives, and the statement that the review does not produce an estimate of the redox effect of levetiracetam.
**METHODS**			
Eligibility criteria	5	Specify the inclusion and exclusion criteria for the review and how studies were grouped for the syntheses.	Section 2.2 (inclusion and exclusion criteria, with the rationale for not restricting the population to epilepsy). Grouping: Section 2.5 (the biomarker-by-comparator cell) and Section 2.6 (Streams A, B and C); Supplementary Table S8.
Information sources	6	Specify all databases, registers, websites, organisations, reference lists and other sources searched or consulted to identify studies. Specify the date when each source was last searched or consulted.	Section 2.3: PubMed/MEDLINE, the Cochrane Library, Web of Science Core Collection and Scopus, each searched from inception through 15 July 2026; Embase not searched, with the reason; reference lists of all included studies and of the reviews retrieved electronically; forward-citation checking in Scopus and Web of Science on 15 July 2026; trial registries, WHO ICTRP, dissertations, preprints and other grey literature not systematically searched. Supplementary Table S7 gives the database of retrieval for every included study.
Search strategy	7	Present the full search strategies for all databases, registers and websites, including any filters and limits used.	Supplementary Table S1 (complete strategies as executed, with coverage dates, for all four databases). Limits are in Section 2.3: English-language full-text articles, prespecified in the registered protocol; no date restriction.
Selection process	8	Specify the methods used to decide whether a study met the inclusion criteria of the review, including how many reviewers screened each record and each report retrieved, whether they worked independently, and if	Section 2.4: two reviewers screened titles and abstracts and assessed full texts independently; unresolved disagreements went to a third reviewer. Section 2.4 also

Section and Topic	Item #	Checklist item	Location where item is reported
		applicable, details of automation tools used in the process.	describes the handling of the two linked companion records (Supplementary Note S3). Any use of generative AI in the review process is declared in Section 2.9.
Data collection process	9	Specify the methods used to collect data from reports, including how many reviewers collected data from each report, whether they worked independently, any processes for obtaining or confirming data from study investigators, and if applicable, details of automation tools used in the process.	Section 2.4: independent extraction using a piloted form, reconciled by consensus after rechecking the source, with a third reviewer for unresolved disagreements; study authors were not contacted, including those of the report discussed in Section 3.4. Any use of generative AI is declared in Section 2.9.
Data items	10a	List and define all outcomes for which data were sought. Specify whether all results that were compatible with each outcome domain in each study were sought (e.g. for all measures, time points, analyses), and if not, the methods used to decide which results to collect.	Section 2.2 (eligible outcome domains: oxidative-stress, antioxidant, redox-related and ferroptosis-related markers). Section 2.5: the synthesis unit is each biomarker result within a defined comparison; multiple results from one cohort were never aggregated and never contributed more than one result to a single biomarker-by-comparator cell; where several follow-up times were reported, the prespecified primary time point was used, or the longest interval where none was prespecified. Supplementary Table S3 lists every result collected.
Data items	10b	List and define all other variables for which data were sought (e.g. participant and intervention characteristics, funding sources). Describe any assumptions made about any missing or unclear information.	Section 2.4 lists the captured items (design, setting, population, age, LEV sample size, dose, duration, comparator, seizure and treatment context, matrix, assay, biomarker values, dispersion, statistical results, attrition, and factors bearing on confounding or measurement validity); Section 2.5 covers matrix. Table 2 and Supplementary Table S2 present these per study, with "NR" where the source does not report the variable. Section 2.4 states that no summary measure was transformed and that graphically presented results were recorded as figure-only rather than estimated from figures; no assumption was substituted for a missing value.
Study risk of bias assessment	11	Specify the methods used to assess risk of bias in the included studies, including details of the tool(s) used, how many reviewers assessed each study and whether they worked independently, and if applicable, details of automation tools used in the process.	Section 2.7: ROBINS-I (2016), applied to the LEV-relevant result of every study against the target trial specified for its stream, with the handling of the "deviations from intended interventions" domain in the absence of formal allocation. Supplementary Table S4 gives all seven domain judgments and the principal driver for each study; Supplementary Note S1 gives the reasoning for not applying a critical judgment. The number of reviewers who assessed each study, and whether they worked independently, is not stated.
Effect measures	12	Specify for each outcome the effect measure(s) (e.g. risk ratio, mean difference) used in the synthesis or presentation of results.	Section 2.6: the prespecified measure was the standardised mean difference with 95% confidence intervals; as reported, between-group contrasts use the mean difference in original units with a 95% confidence interval and Hedges' g, and paired contrasts use the

Section and Topic	Item #	Checklist item	Location where item is reported
			standardised within-person change (dz). Footnote to Table 4 and caption to Figure 2.
Synthesis methods	13a	Describe the processes used to decide which studies were eligible for each synthesis (e.g. tabulating the study intervention characteristics and comparing against the planned groups for each synthesis (item #5)).	Sections 2.5 and 2.6: a result enters a synthesis through its biomarker-by-comparator cell and its comparator stream; the target trial for each stream is specified in Supplementary Table S8. The bodies of evidence formed are listed in Table 5 and Supplementary Table S5.
Synthesis methods	13b	Describe any methods required to prepare the data for presentation or synthesis, such as handling of missing summary statistics, or data conversions.	Section 2.4: no summary measure was transformed (medians were not converted to means, standard errors were not converted to standard deviations, figure-only values were not estimated from figures). Sections 2.6 and 3.3: where the change-score standard deviation was missing, the implied within-person correlation was recovered from an exact reported p value when the implied value fell within the possible range; otherwise intervals are given under assumed correlations of 0.5 and 0.7 and identified as assumption-dependent.
Synthesis methods	13c	Describe any methods used to tabulate or visually display results of individual studies and syntheses.	Section 2.6; Tables 1–5 and Supplementary Tables S2–S5; Figure 2, a forest plot with no pooled estimate, whose caption defines the three panels and the filled/open marker convention.
Synthesis methods	13d	Describe any methods used to synthesise results and provide a rationale for the choice(s). If meta-analysis was performed, describe the model(s), method(s) to identify the presence and extent of statistical heterogeneity, and software package(s) used.	Sections 2.1, 2.6 and 3.3: structured synthesis without meta-analysis, reported following SWiM, because no biomarker-by-comparator cell contained two sufficiently comparable studies with complete variance information. No meta-analysis was performed, so the model, heterogeneity-assessment and software clauses of this item do not apply. The software used to derive the individual estimates in Table 4 is not stated.
Synthesis methods	13e	Describe any methods used to explore possible causes of heterogeneity among study results (e.g. subgroup analysis, meta-regression).	Section 2.6: the five prespecified subgroup analyses (by condition, duration, biomarker class, design and sub-stream) were all infeasible, so the synthesis is structured by comparator sub-stream and biomarker class. Table 2 and Section 3.2 set out the clinical variables that a subgroup analysis would require and show how rarely the sources report them.
Synthesis methods	13f	Describe any sensitivity analyses conducted to assess robustness of the synthesised results.	Section 2.7, consequence (d) of a Level 2 result, fixed before the check was applied; Section 3.9. Supplementary Note S1 sets out the second, stricter reading of Stream A and what changes under it; Supplementary Note S2 gives the operationalisation.
Reporting bias assessment	14	Describe any methods used to assess risk of bias due to missing results in a synthesis (arising from reporting biases).	Section 2.7, final paragraph: qualitative assessment per eligible cell (tabulated, figure-only or unusable result; apparent prespecification; complete reporting of measured biomarkers). Funnel plots and tests for small-study effects were not applicable.

Section and Topic	Item #	Checklist item	Location where item is reported
Certainty assessment	15	Describe any methods used to assess certainty (or confidence) in the body of evidence for an outcome.	Section 2.8: GRADE, applied separately to bodies of evidence defined by comparator stream and outcome domain, starting at low certainty for non-randomised evidence.
**RESULTS**			
Study selection	16a	Describe the results of the search and selection process, from the number of records identified in the search to the number of studies included in the review, ideally using a flow diagram.	Section 3.1 and Figure 1 (PRISMA 2020 flow diagram): 606 records identified; 100 exact duplicates and 2 linked companion records removed; 504 screened; 484 excluded; 20 reports sought for retrieval and all 20 retrieved and assessed; 9 excluded at full text; 11 studies included.
Study selection	16b	Cite studies that might appear to meet the inclusion criteria, but which were excluded, and explain why they were excluded.	Section 3.1 and Supplementary Table S6, which lists all nine reports excluded at full text with journal, DOI and reason. Section 4.5 discusses Ersan et al. and Martinc et al. Supplementary Note S3 documents the two linked companion records and the basis for excluding each.
Study characteristics	17	Cite each included study and present its characteristics.	Section 3.2; Table 1 (design, population, LEV n, exposure, matrix and markers, main finding, main limitation); Table 2 (clinical and treatment context); Supplementary Table S2 (full extraction).
Risk of bias in studies	18	Present assessments of risk of bias for each included study.	Section 3.7; Table 3 (principal concerns and overall judgment for each comparison); Supplementary Table S4 (domain-level judgments D1–D7 and the principal driver for all eleven studies).
Results of individual studies	19	For all outcomes, present, for each study: (a) summary statistics for each group (where appropriate) and (b) an effect estimate and its precision (e.g. confidence/credible interval), ideally using structured tables or plots.	(a) Supplementary Table S3, which reproduces the comparator/baseline and LEV/follow-up values as published. (b) Table 4 and Figure 2 give an effect estimate with a 95% confidence interval for every result from which one could be derived; Panel C of Figure 2 and the foot of Table 4 name the four studies, and the one further outcome, from which none could, with the reason in each case. Sections 3.4–3.6 present them by stream.
Results of syntheses	20a	For each synthesis, briefly summarise the characteristics and risk of bias among contributing studies.	The opening paragraph of each of Sections 3.4, 3.5 and 3.6 states the risk-of-bias verdict for the whole stream before any number is given. Table 5 and Supplementary Table S5 give, for each body of evidence, the number of studies, the participants contributing LEV data and the domain-level certainty judgments.
Results of syntheses	20b	Present results of all statistical syntheses conducted. If meta-analysis was done, present for each the summary estimate and its precision (e.g. confidence/credible interval) and measures of statistical heterogeneity. If comparing groups, describe the direction of the effect.	Sections 3.4–3.6; Table 4; Figure 2. No meta-analysis was performed and no pooled estimate is presented, so no summary estimate or heterogeneity statistic is reported. The direction of each result is described in the text and encoded in Figure 2, whose header states the sign convention for damage and antioxidant markers.
Results of syntheses	20c	Present results of all investigations of possible causes of heterogeneity among study results.	Section 3.2 reports the principal result of Table 2: the clinical variables that would explain divergence between cohorts are

Section and Topic	Item #	Checklist item	Location where item is reported
			largely not recoverable from the sources. Section 4.2 discusses the refractory-versus-drug-naive contrast on that basis. Section 2.6 records that the five prespecified subgroup analyses were infeasible.
Results of syntheses	20d	Present results of all sensitivity analyses conducted to assess the robustness of the synthesised results.	Section 3.9: ten studies and 265 participants remain; no certainty rating changes and no interval in Table 4 changes; the ferroptosis-related body disappears entirely. The “Studies (sensitivity analysis)” column of Table 5 gives the count for each body. Supplementary Note S1 reports the second, stricter reading of Stream A.
Reporting biases	21	Present assessments of risk of bias due to missing results (arising from reporting biases) for each synthesis assessed.	Section 3.7: selective reporting could not be excluded in ten of the eleven studies, with the single exception and its basis stated. Domain D7 of Supplementary Table S4; the “Publication and reporting bias” column of Supplementary Table S5, per body of evidence; Section 4.6.
Certainty of evidence	22	Present assessments of certainty (or confidence) in the body of evidence for each outcome assessed.	Section 3.8; Table 5; Supplementary Table S5 (full evidence profile with the domain-level downgrading judgments).
**DISCUSSION**			
Discussion	23a	Provide a general interpretation of the results in the context of other evidence.	Sections 4.1 and 4.3; Section 4.5 relates the findings to the experimental literature and to two human reports that provide no LEV-specific estimate.
Discussion	23b	Discuss any limitations of the evidence included in the review.	Sections 4.1, 4.2 and 4.3; Section 4.6, second paragraph; Table 3 and Table 5.
Discussion	23c	Discuss any limitations of the review processes used.	Section 4.6, third paragraph: Embase not searched and why that matters here (Supplementary Table S7); trial registries and grey literature not systematically searched; the prespecified English-language restriction and the unpredictable direction of the resulting bias; selective-outcome and publication bias not assessable statistically.
Discussion	23d	Discuss implications of the results for practice, policy, and future research.	Section 4.4 (why oxidative biomarkers cannot at present guide levetiracetam prescribing or monitoring); Section 4.7 (research recommendations, including the conditions under which individualised redox-informed treatment selection would become defensible); Section 5.
**OTHER INFORMATION**			
Registration and protocol	24a	Provide registration information for the review, including register name and registration number, or state that the review was not registered.	Section 2.1 and the Abstract: PROSPERO, CRD420261448016, registered before study selection.
Registration and protocol	24b	Indicate where the review protocol can be accessed, or state that a protocol was not prepared.	Section 2.1 and the Data Availability Statement: Open Science Framework, doi:10.17605/OSF.IO/HXA4U.
Registration and protocol	24c	Describe and explain any amendments to information provided at registration or in the protocol.	Section 2.1: no material deviation from the prespecified eligibility criteria; two included studies contributed results from matrices beyond those listed in the protocol (Section 2.5); two additions were made after registration and are identified as post hoc

Section and Topic	Item #	Checklist item	Location where item is reported
			throughout — the source-verifiability and reporting-consistency check of Section 2.7 and the sensitivity analysis of Section 3.9.
Support	25	Describe sources of financial or non-financial support for the review, and the role of the funders or sponsors in the review.	Funding: "This research received no external funding."
Competing interests	26	Declare any competing interests of review authors.	Conflicts of Interest: "The authors declare no conflict of interest."
Availability of data, code and other materials	27	Report which of the following are publicly available and where: template data collection forms; data extracted from included studies; data used for all analyses; analytic code; any other materials used in the review.	Data Availability Statement. Supplementary Material 2 contains the search strategies (Table S1), the data extracted from the included studies (Tables S2 and S3), the data used for all analyses (Table S3), the risk-of-bias and certainty assessments (Tables S4 and S5), the reasons for exclusion at full text (Table S6), the database of retrieval (Table S7), the target-trial specifications (Table S8) and Notes S1–S3. The protocol is on OSF (doi:10.17605/OSF.IO/HXA4U). The template data-collection form and the analytic code used to derive the estimates in Table 4 are not separately deposited; both are offered on reasonable request under the Data Availability Statement.

Table B. PRISMA 2020 for Abstracts checklist.

Section and Topic	Item #	Checklist item	Location where item is reported
**TITLE**			
Title	1	Identify the report as a systematic review.	Title ("… A Systematic Review of Human Evidence"); article-type line ("Systematic review").
**BACKGROUND**			
Objectives	2	Provide an explicit statement of the main objective(s) or question(s) the review addresses.	Abstract: "We systematically reviewed human evidence on levetiracetam (LEV) under three estimands: within-person change, LEV-treated epilepsy versus healthy controls, and LEV versus other antiseizure medications."
**METHODS**			
Eligibility criteria	3	Specify the inclusion and exclusion criteria for the review.	Partly. The Abstract states the population, the exposure and the three comparators ("human evidence on levetiracetam (LEV) under three estimands …") and the design of the included studies ("Eleven non-randomized studies …"), but the formal inclusion and exclusion criteria are not restated there because of the 200-word limit. They are in Section 2.2.
Information sources	4	Specify the information sources (e.g. databases, registers) used to identify studies and the date when each was last searched.	Abstract: "PubMed/MEDLINE, Cochrane Library, Web of Science and Scopus were searched from inception to 15 July 2026 …"
Risk of bias	5	Specify the methods used to assess risk of bias in the included studies.	Not stated in the Abstract. Risk of bias was assessed with ROBINS-I (Section 2.7; Supplementary Table S4).
Synthesis of results	6	Specify the methods used to present and synthesise results.	Abstract: "Effect estimates with 95% confidence intervals were calculated here

Section and Topic	Item #	Checklist item	Location where item is reported
			wherever reporting permitted …" and "Meta-analysis was inappropriate: definitions, matrices, assays, estimands and variance reporting were incompatible."
**RESULTS**			
Included studies	7	Give the total number of included studies and participants and summarise relevant characteristics of studies.	Abstract: "Eleven non-randomized studies contributed 297 participants — the sum of LEV subgroups with analysable biomarker data, not the total enrolled populations."
Synthesis of results	8	Present results for main outcomes, preferably indicating the number of included studies and participants for each. If meta-analysis was done, report the summary estimate and confidence/credible interval. If comparing groups, indicate the direction of the effect (i.e. which group is favoured).	Abstract: "… none could be derived for four studies, and those derived are wide, inconsistent in direction, or from designs that cannot attribute change to LEV." No meta-analysis was done, so no summary estimate is reported; no direction of effect is claimed, because none is established. Per-outcome numbers of studies and participants are given in Table 5 and Supplementary Table S5 rather than in the Abstract.
**DISCUSSION**			
Limitations of evidence	9	Provide a brief summary of the limitations of the evidence included in the review (e.g. study risk of bias, inconsistency and imprecision).	Abstract: "… wide, inconsistent in direction, or from designs that cannot attribute change to LEV" and "Certainty was very low for every outcome-domain/comparator body …"
Interpretation	10	Provide a general interpretation of the results and important implications.	Abstract, final sentence: "This review's contribution is not an estimate of LEV's redox effect but a demonstration of why the literature cannot establish one: the human evidence is not of sufficient validity to support or refute such an effect."
**OTHER**			
Funding	11	Specify the primary source of funding for the review.	Not stated in the Abstract. Declared under Funding: "This research received no external funding."
Registration	12	Provide the register name and registration number.	Abstract: "(PROSPERO CRD420261448016; OSF doi:10.17605/OSF.IO/HXA4U)".

.
