# Supplementary material for: Levetiracetam and Peripheral Oxidative Stress Biomarkers in Epilepsy: A Systematic Review of Human Evidence
Source: Int J Mol Sci. 2026 Sep 21;27(18):8410. doi: 10.3390/ijms27188410 (PMC13607205; doi:10.3390/ijms27188410)
Supplement: Supplementary file 1 [file ijms-27-08410-s001.zip › Supplementary_Material_2_R1.pdf]

Supplementary Material

Levetiracetam and Peripheral Oxidative-Stress Biomarkers in Epilepsy: A Systematic Review of Human Evidence

Table S1. Complete electronic search strategies

Database-specific search strategies as executed on 15 July 2026.

Database	Coverage	Complete strategy
PubMed/MEDLINE	Inception to 15 July 2026	("Levetiracetam"[MeSH Terms] OR levetiracetam[tiab] OR keppra[tiab] OR "UCB-L059"[tiab] OR "UCB L059"[tiab] OR etiracetam[tiab]) AND ("Oxidative Stress"[MeSH Terms] OR "Reactive Oxygen Species"[MeSH Terms] OR "Lipid Peroxidation"[MeSH Terms] OR "Free Radicals"[MeSH Terms] OR "Glutathione"[MeSH Terms] OR "Superoxide Dismutase"[MeSH Terms] OR "Catalase"[MeSH Terms] OR "Malondialdehyde"[MeSH Terms] OR "8-Hydroxy-2'-Deoxyguanosine"[MeSH Terms] OR "Isoprostanes"[MeSH Terms] OR "Antioxidants"[MeSH Terms] OR "oxidative stress"[tiab] OR "reactive oxygen species"[tiab] OR ROS[tiab] OR "free radical*"[tiab] OR "lipid peroxidation"[tiab] OR malondialdehyde[tiab] OR MDA[tiab] OR TBARS[tiab] OR "thiobarbituric acid reactive substances"[tiab] OR "superoxide dismutase"[tiab] OR SOD[tiab] OR catalase[tiab] OR glutathione[tiab] OR GSH[tiab] OR GSSG[tiab] OR "glutathione peroxidase"[tiab] OR GPx[tiab] OR "8-hydroxy-2-deoxyguanosine"[tiab] OR "8-OHdG"[tiab] OR "8-hydroxydeoxyguanosine"[tiab] OR isoprostane*[tiab] OR "4-hydroxynonenal"[tiab] OR "4-HNE"[tiab] OR "protein carbonyl*"[tiab] OR "oxidative damage"[tiab] OR "nitric oxide"[tiab] OR peroxynitrite[tiab] OR "total antioxidant capacity"[tiab] OR TAC[tiab] OR "total oxidant status"[tiab] OR TOS[tiab] OR "oxidative stress index"[tiab] OR OSI[tiab] OR thioredoxin[tiab] OR paraoxonase[tiab] OR "glutathione reductase"[tiab] OR antioxidant*[tiab] OR "redox status"[tiab] OR "oxidant stress"[tiab]) AND "Humans"[MeSH Terms]
Cochrane Library	Inception to 15 July 2026	(MeSH descriptor: [Levetiracetam] explode all trees OR levetiracetam:ti,ab,kw OR keppra:ti,ab,kw OR "UCB-L059":ti,ab,kw OR etiracetam:ti,ab,kw) AND (MeSH descriptor: [Oxidative Stress] explode all trees OR MeSH descriptor: [Reactive Oxygen Species] explode all trees OR MeSH descriptor: [Lipid Peroxidation] explode all trees OR MeSH descriptor: [Free Radicals] explode all trees OR MeSH descriptor: [Glutathione] explode all trees OR MeSH descriptor: [Superoxide Dismutase] explode all trees OR MeSH descriptor: [Catalase] explode all trees OR MeSH descriptor: [Malondialdehyde] explode all trees OR MeSH descriptor: [8-Hydroxy-2'-Deoxyguanosine] explode all trees OR MeSH descriptor: [Isoprostanes] explode all trees OR MeSH descriptor: [Antioxidants] explode all trees OR "oxidative stress":ti,ab,kw OR "reactive oxygen species":ti,ab,kw OR ROS:ti,ab,kw OR "free radical*":ti,ab,kw OR "lipid peroxidation":ti,ab,kw OR malondialdehyde:ti,ab,kw OR MDA:ti,ab,kw OR TBARS:ti,ab,kw OR "superoxide dismutase":ti,ab,kw OR SOD:ti,ab,kw OR catalase:ti,ab,kw OR glutathione:ti,ab,kw OR GSH:ti,ab,kw OR GSSG:ti,ab,kw OR "glutathione peroxidase":ti,ab,kw OR GPx:ti,ab,kw OR "8-hydroxy-2-deoxyguanosine":ti,ab,kw OR "8-OHdG":ti,ab,kw OR isoprostane*:ti,ab,kw OR "4-hydroxynonenal":ti,ab,kw OR "protein carbonyl*":ti,ab,kw OR "oxidative damage":ti,ab,kw OR "nitric oxide":ti,ab,kw OR peroxynitrite:ti,ab,kw OR "total antioxidant capacity":ti,ab,kw OR TAC:ti,ab,kw OR "total oxidant status":ti,ab,kw OR TOS:ti,ab,kw OR "oxidative stress index":ti,ab,kw OR OSI:ti,ab,kw OR thioredoxin:ti,ab,kw OR paraoxonase:ti,ab,kw OR "glutathione reductase":ti,ab,kw OR antioxidant*:ti,ab,kw OR "redox status":ti,ab,kw)
Web of Science Core Collection	Inception to 15 July 2026	TS=(levetiracetam OR keppra OR "UCB-L059" OR "UCB L059" OR etiracetam) AND TS=("oxidative stress" OR "reactive oxygen species" OR ROS OR "free radical*" OR "lipid peroxidation" OR malondialdehyde OR MDA OR TBARS OR "thiobarbituric acid reactive substances" OR "superoxide dismutase" OR SOD OR catalase OR glutathione OR GSH OR GSSG OR "glutathione peroxidase" OR GPx OR "8-hydroxy-2-deoxyguanosine" OR "8-OHdG" OR "8-hydroxydeoxyguanosine" OR isoprostane* OR "4-hydroxynonenal" OR "4-HNE" OR "protein carbonyl*" OR "oxidative damage" OR "nitric oxide" OR peroxynitrite OR "total antioxidant capacity" OR TAC OR "total oxidant status" OR TOS OR "oxidative stress index" OR OSI OR thioredoxin OR paraoxonase OR "glutathione reductase" OR antioxidant* OR "redox status" OR "oxidant stress") AND TS=(human* OR patient* OR clinical OR "clinical trial*" OR cohort* OR "case-control" OR randomized OR randomised OR "cross-sectional")
Scopus	Inception to 15 July 2026	TITLE-ABS-KEY(levetiracetam OR keppra OR "UCB-L059" OR "UCB L059" OR etiracetam) AND TITLE-ABS-KEY("oxidative stress" OR "reactive oxygen species" OR ROS OR "free radical*" OR "lipid peroxidation" OR malondialdehyde OR MDA OR TBARS OR "thiobarbituric acid reactive substances" OR "superoxide dismutase" OR SOD OR catalase OR glutathione OR GSH OR GSSG OR "glutathione peroxidase" OR GPx OR "8-hydroxy-2-deoxyguanosine" OR "8-OHdG" OR "8-hydroxydeoxyguanosine" OR isoprostane* OR "4-hydroxynonenal" OR "4-HNE" OR "protein carbonyl*" OR "oxidative damage" OR "nitric oxide" OR peroxynitrite OR "total antioxidant capacity" OR TAC OR "total oxidant status" OR TOS OR "oxidative stress index" OR OSI OR thioredoxin OR paraoxonase OR "glutathione reductase" OR antioxidant* OR "redox status" OR "oxidant stress") AND TITLE-ABS-KEY(human* OR patient* OR clinical OR "clinical trial*" OR cohort* OR "case-control" OR randomized OR randomised OR "cross-sectional")

Table S2. Characteristics of included studies

Design, population, exposure, and biomarker characteristics of the 11 included studies. The column "LEV n" gives the number of LEV-treated participants with analysable biomarker data, which is the denominator used throughout the review and sums to 297. ARE, arylesterase; ASM, antiseizure medication; CBZ, carbamazepine; GPx, glutathione peroxidase; GPX4, glutathione peroxidase 4; GSH, glutathione; IQR, interquartile range; LEV, levetiracetam; MDA, malondialdehyde; NO, nitric oxide; NR, not reported; NS, not significant; 8-OHdG, 8-hydroxy-2'-deoxyguanosine; 8-OHG, 8-hydroxyguanine; ox-LDL, oxidized low-density lipoprotein; PON1, paraoxonase-1; RoB, risk of bias; SD, standard deviation; SEM, standard error of the mean; SLC7A11, solute carrier family 7 member 11; SOD, superoxide dismutase; TAC, total antioxidant capacity; VPA, valproate.

Study	Country	Design	Comparator stream	Population	Age group	LEV n	Total N context	LEV dose	LEV duration	Matrix	Markers	Direction	Numeric availability	Overall RoB	DOI
Varoglu 2010	Turkey	Cross-sectional	B: LEV-treated vs healthy controls	Epilepsy, age 17-56 years	Adults	8	61 patients stated + 30 controls; group counts conflict	NR	LEV-specific NR; ASM therapy overall mean 3.90 +/- 5.76 y	Serum; leukocyte DNA	PON1, ARE, ox-LDL, 8-OHG	Pro-oxidant for ARE and 8-OHG; PON1/ox-LDL not significant	LEV subgroup means only; no SD	Serious	10.1097/WNF.0b013e3181d1e133
Ozden 2010	Turkey	Prospective single-arm pre-post	A: baseline vs 3-month LEV	Cryptogenic partial epilepsy; mean age 29.6 y	Adults	21	30 considered; 21 analyzed	1000-3000 mg/day	3 months	Urine	15-F2t-isoprostane	Pro-oxidant: increased after LEV (p=0.025)	Mean +/- SD; unit reported inconsistently	Serious	10.1016/j.seizure.2010.07.010
Chen 2015	China	Prospective single-arm pre-post	A: before vs after LEV; interval NR	Refractory epilepsy, age 16-63 y	Adolescents/adults	72	72	10 mg/kg BID initially; 20-40 mg/kg/day	NR	Serum	PON1, ARE, ox-LDL	Pro-oxidant: PON1/ARE down and ox-LDL up (all p<0.05)	Mean +/- SD	Serious	10.1007/s12013-015-0683-8
Elsayed/Shatla 2016	Egypt	Prospective cohort; pooled pre-post and post-treatment active-ASM comparison	C: LEV vs other ASMs (LEV-specific); pooled pre-post is not LEV-specific	Newly diagnosed idiopathic epilepsy	Children	14	110 initially; 84 completed + 42 controls	NR	6 months	Serum/plasma	MDA, NO, GPx, SOD, catalase	LEV-specific change indeterminate; post-treatment GPx lowest with LEV (p=0.046), other markers NS	LEV post-treatment mean +/- SD only	Serious	10.1055/s-0036-1586728
Haznedar 2019	Turkey	Cross-sectional	B: LEV-treated vs healthy controls	Generalized epilepsy, age >2 y	Children	26	26 LEV + 25 VPA + 26 controls	20-60 mg/kg/day	>=6 months	Serum	MDA, 8-OHdG	Pro-oxidant: MDA and 8-OHdG higher vs controls	Values/dispersion only in figures	Serious	10.1016/j.eplepsyres.2019.03.009
Mahdavi 2020	Iran	Case-control, cross-sectional	B: LEV-treated vs healthy controls	Generalized or focal epilepsy, age 18-60 y	Adults	33	33 LEV + 35 controls	Mean 992.42 +/- 477.97 mg/day	>=6 months; mean 2.24 +/- 2.21 y	Serum; saliva	TAC, SOD	Pro-oxidant for TAC (lower, p=0.02); SOD neutral	Mean +/- SD	Serious	10.4314/ahs.v20i2.55
Sarangi 2019	India	Cross-sectional observational	C: LEV vs conventional ASMs	Epilepsy, age >18 y	Adults	36	163 overall; 58 LEV-exposed; biomarker subset 74	1000-3000 mg/day	>=6 months	Serum	TAC	No difference detected between LEV and conventional ASMs within the subgroup positive for psychiatric/behavioural adverse effects; the source reports no TAC value for either group as a whole	Medians/IQR in selected subset	Serious	10.1016/j.yebeh.2019.07.040
Coban Ramazan 2019	Turkey	Cross-sectional	B: LEV-treated vs	Idiopathic epilepsy, age 15-65 y	Adolescents/adults	13	47 patients + 38 controls	NR	Mean 7.25 +/- 5.47 y	Plasma	GSH, cysteine, cysteinylglycine, homocysteine	Neutral for LEV	Mean +/- SD	Serious	10.1515/tjb-2018-0218

Study	Country	Design	Comparator stream	Population	Age group	LEV n	Total N context	LEV dose	LEV duration	Matrix	Markers	Direction	Numeric availability	Overall RoB	DOI
			healthy controls												
Mehvari-Habibabadi 2022	Iran	Prospective single-arm pre-post	A: baseline vs 3-month LEV	Epilepsy, age 16-68 y	Adolescents/adults	30	50 approached; 34 entered follow-up; 30 completed	1000-3000 mg/day	3 months	Serum	GSH, zinc	No clear antioxidant effect: GSH not interpretable (printed change statistics irreconcilable); zinc decreased but significance internally inconsistent	Mean +/- SD; unit for zinc unclear in the source report	Serious	10.18502/cjn.v21i4.11719
Swathi/Aruna 2022	India	Open-label non-randomized pre-post	A: drug-naive baseline vs 3-month LEV	Newly diagnosed drug-naive epilepsy, age 18-60 y	Adults	12	45 enrolled across ASMs (15 assigned LEV); 39 analyzed	500 mg twice daily	3 months	Serum	MDA, NO, GSH	Protective: MDA down, GSH up; NO NS	Mean +/- SD	Serious	10.7860/JCDR/2022/57376.17106
Wang 2025	China	Prospective non-randomized pre-post with healthy controls and active-ASM groups	A: LEV before-after; healthy and active comparators contextual	First-time-medication epilepsy, age 18-60 y	Adults	32	98 patients enrolled + 93 controls; 95 patients analyzed	Monotherapy per label; dose NR	NR	Plasma/blood; leukocyte RNA/protein	Fe2+, MDA, GSH, GPX4, SLC7A11	Protective: Fe2+/MDA down; GSH/GPX4/SLC7A11 up	Figure-only, mean +/- SEM	Serious	10.1016/j.jrras.2025.101558

Table S3. Numeric results

Reported comparator/baseline and levetiracetam/follow-up values with inference and qualifications, exactly as published in the source reports. Values presented only graphically in the source are identified as such and were not estimated from the figures. ARE, arylesterase; ASM, antiseizure medication; CBZ, carbamazepine; GPx, glutathione peroxidase; GPX4, glutathione peroxidase 4; GSH, glutathione; IQR, interquartile range; LEV, levetiracetam; MDA, malondialdehyde; NO, nitric oxide; NR, not reported; NS, not significant; 8-OHdG, 8-hydroxy-2'-deoxyguanosine; 8-OHG, 8-hydroxyguanine; ox-LDL, oxidized low-density lipoprotein; PON1, paraoxonase-1; RoB, risk of bias; SD, standard deviation; SEM, standard error of the mean; SLC7A11, solute carrier family 7 member 11; SOD, superoxide dismutase; TAC, total antioxidant capacity; VPA, valproate.

Study	Principal limitation on interpretation	Outcome	Comparator/baseline	LEV/follow-up	Reported inference
Varoglu 2010	Means without LEV subgroup SD; source table marks these only as p<0.05 and p<0.0001	ARE; 8-OHG	99.75 U/mL; 0.41/10^5 guanine	62.70 U/mL; 1.36/10^5 guanine	ARE p=0.017; 8-OHG p=0.0001 (LEV subgroup, n=8, vs controls)
Ozden 2010	Unit inconsistent within source; paired-change SD absent	15-F2t-isoprostane	876 ± 447	1,560 ± 630	p=0.025
Chen 2015	Follow-up duration and co-treatment unclear	PON1; ARE; ox-LDL	132.79 ± 18.36; 99.17 ± 5.21; 115.69 ± 13.16	105.29 ± 8.27; 72.08 ± 4.59; 150.29 ± 16.25	all p<0.05
Haznedar 2019	Exact means/dispersion not tabulated	MDA; 8-OHdG	Healthy-control values in figures	LEV values in figures	p<0.001; p=0.018
Mahdavi 2020	Cross-sectional and diet-confounded	TAC; salivary SOD	19.0 ± 7.8; 32.4 ± 8.9	15.8 ± 5.2; 31.7 ± 8.5	p=0.02; p=0.75
Elsayed 2016	No LEV-specific baseline; VPA also in comparison	Post-treatment MDA; GPx	CBZ 4.6 ± 2.4; 135.7 ± 16.3	LEV 6.5 ± 2.9; 123.9 ± 7.3	across drugs p=0.078; p=0.046
Çoban Ramazan 2019	Peak separation problem	GSH; cysteine	2.42 ± 2.53; 171.44 ± 43.86	3.54 ± 1.84; 146.86 ± 40.17	LEV-control differences not significant
Mehvari-Habibabadi 2022	Zinc p, t, CI, and narrative conflict; glutathione change	GSH; zinc	381.60 ± 135.19; 80.08 ± 18.33	349.35 ± 159.77; 73.46 ± 18.88	GSH p=0.068; zinc unresolved

Study	Principal limitation on interpretation	Outcome	Comparator/baseline	LEV/follow-up	Reported inference
	(-66.78) irreconcilable with its own means and t				
Swathi and Aruna 2022	n=12; paired-change SD absent	MDA; NO; GSH	4.51 ± 1.61; 49.24 ± 13.03; 447.52 ± 105.1	4.02 ± 1.34; 42.37 ± 12.11; 522.96 ± 121.2	p=0.0338; p=0.0791; p<0.0001
Wang 2025	Exact values not tabulated; mean ± SEM	Fe2+, MDA, GSH, GPX4, SLC7A11	Pretreatment figure values	Post-treatment figure values	Reported favorable changes
Sarangi 2019	The source prints no TAC value for the LEV group (36) or the conventional group (38) as a whole; 0.610 is the only LEV-versus-conventional p value given and it compares PBAE-positive subgroups only. Medians and IQRs were not converted (Section 2.4), so no standardized estimate is available.	TAC	Conventional ASMs, PBAE-positive subgroup (n=13): median 398.88 (IQR 348.11–434.11)	LEV, PBAE-positive subgroup (n=23): median 411.77 (IQR 265.27–515.27)	p=0.610

Table S4. ROBINS-I risk-of-bias assessment

Domain-level (D1–D7) and overall risk-of-bias judgments with main drivers, following the 2016 ROBINS-I framework. D1, confounding; D2, selection of participants; D3, classification of interventions; D4, deviations from intended interventions; D5, missing data; D6, measurement of outcomes; D7, selection of the reported result. ARE, arylesterase; ASM, antiseizure medication; CBZ, carbamazepine; GPx, glutathione peroxidase; GPX4, glutathione peroxidase 4; GSH, glutathione; IQR, interquartile range; LEV, levetiracetam; MDA, malondialdehyde; NO, nitric oxide; NR, not reported; NS, not significant; 8-OHdG, 8-hydroxy-2'-deoxyguanosine; 8-OHG, 8-hydroxyguanine; ox-LDL, oxidized low-density lipoprotein; PON1, paraoxonase-1; RoB, risk of bias; SD, standard deviation; SEM, standard error of the mean; SLC7A11, solute carrier family 7 member 11; SOD, superoxide dismutase; TAC, total antioxidant capacity; VPA, valproate.

Study	D1 Confounding	D2 Selection	D3 Classification	D4 Deviations	D5 Missing data	D6 Outcome measurement	D7 Reported result	Overall	Main driver
Varoglu 2010	Serious	Moderate	Moderate	Moderate	Low	Moderate	Serious	Serious	Epilepsy/duration confounding; subgroup dispersion absent; denominator inconsistency.
Ozden 2010	Serious	Moderate	Low	Moderate	Moderate	Low	Low	Serious	Uncontrolled pre-post comparison; exclusion based on recent seizures.
Chen 2015	Serious	Moderate	Moderate	Serious	Moderate	Moderate	Moderate	Serious	Uncontrolled pre-post comparison; duration and concomitant treatment unclear.
Elsayed/Shatla 2016	Serious	Moderate	Moderate	Moderate	Serious	Moderate	Serious	Serious	No LEV-specific baseline; unexplained attrition; selective LEV reporting.
Haznedar 2019	Serious	Moderate	Low	Moderate	Low	Moderate	Serious	Serious	Disease confounding; values/dispersion only in figures.
Mahdavi 2020	Serious	Moderate	Low	Moderate	Low	Moderate	Moderate	Serious	Disease and diet confounding; no pre-LEV data.
Sarangi 2019	Serious	Moderate	Low	Moderate	Moderate	Moderate	Serious	Serious	Confounding by indication; biomarker subset and incomplete group-level reporting.
Coban Ramazan 2019	Serious	Moderate	Moderate	Moderate	Low	Serious	Moderate	Serious	Disease confounding; chromatographic peak non-separation.
Mehvari-Habibabadi 2022	Serious	Moderate	Low	Low	Moderate	Moderate	Serious	Serious	Uncontrolled pre-post design; inconsistent zinc statistics/reporting.

Study	D1 Confounding	D2 Selection	D3 Classification	D4 Deviations	D5 Missing data	D6 Outcome measurement	D7 Reported result	Overall	Main driver
Swathi/Aruna 2022	Serious	Moderate	Low	Moderate	Moderate	Low	Moderate	Serious	Non-random treatment and no untreated control; incomplete LEV analysis.
Wang 2025	Serious	Moderate	Moderate	Moderate	Low	Moderate	Serious	Serious	Uncontrolled before-after design with no concurrent untreated epilepsy comparator; dose/duration absent; figure-only outcomes reported as mean +/- SEM; unresolved discrepancies between the stated methods and the reported content (Note S2).

Table S5. GRADE evidence profile

Certainty of evidence by comparator stream and outcome domain, with domain-level downgrading judgments. Every body of evidence consists exclusively of non-randomized studies and therefore started at low certainty; no upgrading criterion (large magnitude of effect, dose-response gradient, or plausible residual confounding working against the observed effect) was met in any body. No body provided a common effect estimate, so the effect column reports the range of individual study results rather than a pooled magnitude. Judgments are those of the review authors. ARE, arylesterase; ASM, antiseizure medication; CBZ, carbamazepine; GPx, glutathione peroxidase; GPX4, glutathione peroxidase 4; GSH, glutathione; IQR, interquartile range; LEV, levetiracetam; MDA, malondialdehyde; NO, nitric oxide; NR, not reported; NS, not significant; 8-OHdG, 8-hydroxy-2'-deoxyguanosine; 8-OHG, 8-hydroxyguanine; ox-LDL, oxidized low-density lipoprotein; PON1, paraoxonase-1; RoB, risk of bias; SD, standard deviation; SEM, standard error of the mean; SLC7A11, solute carrier family 7 member 11; SOD, superoxide dismutase; TAC, total antioxidant capacity; VPA, valproate.

Outcome domain and comparator stream	No. of studies (design)	Participants contributing LEV data	Risk of bias	Inconsistency	Indirectness	Imprecision	Publication and reporting bias	Summary of effect	Certainty
Within-person change: oxidative molecular damage	4 (non-randomized: 4 uncontrolled before-after)	137 (Ozden 21; Chen 72; Swathi and Aruna 12; Wang 32)	Serious: all four studies rated serious overall on ROBINS-I (Table S4); no concurrent untreated epilepsy comparator; Ozden selected on recent seizures; Chen follow-up interval and co-medication unreported	Serious: direction opposed across cohorts (directions do not converge across the four cohorts; results within each cohort are statistically dependent and are not counted)	Not serious for the biomarker question; peripheral damage products are the stated outcome	Serious: 12-72 participants per study; no confidence intervals; paired change-score variance reported by one study only, in a form not reconcilable with its own summary statistics	Serious: no prespecified primary outcome or public analysis plan in any study but Swathi and Aruna, for which a registry record exists; Wang reports outcomes only as figures with uniform significance thresholds	No pooled estimate. Ozden urinary 15-F2t-isoprostane +78%; Chen ox-LDL +30%; Swathi and Aruna MDA -11%; Wang MDA reported as decreased (figure only)	Very low
Within-person change: antioxidant defense and redox substrates	4 (non-randomized: 4 uncontrolled before-after)	146 (Chen 72; Mehvari-Habibabadi 30; Swathi and Aruna 12; Wang 32)	Serious: all four rated serious overall on ROBINS-I; Mehvari-Habibabadi reports internally	Serious: PON1 and arylesterase fell in Chen, glutathione was not interpretable in Mehvari-	Serious: circulating enzyme activity and glutathione are indirect surrogates for tissue redox state and may	Serious: 12-72 participants per study; no confidence intervals; paired change-score variance reported by one study only, and not	Serious: multiple biomarkers measured without prespecified primary outcomes in all but one study; figure-only	No pooled estimate. Chen PON1 -21% and arylesterase -27%; Mehvari-Habibabadi	Very low

			irreconcilable zinc and glutathione statistics	Habibabadi, and glutathione rose in Swathi and Aruna and in Wang	rise adaptively or fall through depletion	reconcilable with its own summary statistics	reporting in Wang	zinc -8% (mean change -6.68, 95% CI -11.95 to -1.41), glutathione not interpretable ; Swathi and Aruna glutathione +17%	
LEV-treated epilepsy versus healthy controls: molecular damage	2 (non-randomized: 2 cross-sectional)	34 (Varoglu 8; Haznedar 26)	Serious: both rated serious overall; Varoglu reports no LEV-subgroup dispersion and has an unexplained denominator inconsistency; Haznedar reports key outcomes only in figures	Not serious: both studies point in the same direction	Very serious: the contrast is LEV-treated epilepsy versus no epilepsy, so treatment status is structurally confounded with disease and recent seizure activity; it cannot identify a medication effect	Serious: 8 and 26 LEV participants ; no dispersion for the Varoglu LEV subgroup	Serious: selective reporting cannot be excluded; no prespecified primary outcome	No pooled estimate. Varoglu 8-OHG 1.36 vs 0.41 per 10^5 guanine (p=0.0001); Haznedar MDA p<0.001 and 8-OHdG p=0.018 versus controls	Very low
LEV-treated epilepsy versus healthy controls: antioxidant capacity and aminothiols	3 (non-randomized: 3 cross-sectional)	54 (Varoglu 8; Mahdavi 33; Coban Ramazan 13)	Serious: all three rated serious overall; incomplete chromatographi c peak separation in Coban Ramazan; dietary	Serious: total antioxidant capacity was lower in Mahdavi, arylesterase was lower in Varoglu, but aminothiols did not differ	Serious: same structural disease confounding as the damage domain, compounded by diet and other between-	Serious: 8-33 LEV participants per study; wide dispersion; no confidence intervals for between-	Serious: multiple aminothiols measured without a prespecified primary outcome	No pooled estimate. Varoglu arylesterase 62.70 vs 99.75 U/mL (p=0.017); Mahdavi TAC 15.8 +/- 5.2 versus 19.0	Very low

			imbalance in Mahdavi	in Coban Ramazan	group differences	group differences		+/- 7.8 (p=0.02); salivary SOD not different (p=0.75)	
LEV versus other antiseizure medications: antioxidant and damage markers	4 (non-randomized: 2 cross-sectional, 2 prospective cohort)	108 (Elsayed 14; Haznedar 26; Sarangi 36; Wang 32)	Serious: all four rated serious overall; Elsayed has no LEV-specific baseline and unexplained attrition; Sarangi reports a selected biomarker subset	Serious: higher MDA than valproate in Haznedar, lowest GPx with LEV in Elsayed, no TAC difference in Sarangi, and a favourable pattern in Wang	Serious: comparators are heterogeneous and partly pooled (Sarangi combines carbamazepine, phenytoin and valproate), so the contrast is relative rather than absolute and is open to confounding by indication	Serious: 14-36 LEV participants per comparison; no confidence intervals	Serious: figure-only reporting in Haznedar and Wang; group-level values incompletely reported in Sarangi	No pooled estimate. Elsayed GPx across drugs p=0.046 and MDA p=0.078; Haznedar MDA higher than valproate p=0.002; Sarangi TAC p=0.610	Very low
Within-person change: ferroptosis-related peripheral markers	1 (non-randomized: 1 uncontrolled before-after)	32 (Wang 32)	Very serious: single study rated serious overall on ROBINS-I; its nominal principal method (thermal infrared imaging) does not appear in the Methods or Results and generates no reported data; all biomarker outcomes are reported only as figure-based means with standard errors	Not assessable: single study	Serious: Fe2+, GPX4 and SLC7A11 measured in peripheral blood do not demonstrate neuronal ferroptosis or its inhibition	Serious: 32 participants; values available only as figure-based means with standard errors	Serious: all outcomes reported graphically with uniform significance thresholds; no prespecified primary outcome	No usable estimate. Fe2+ and MDA reported as decreased and GSH, GPX4 and SLC7A11 as increased, all from figures	Very low

Table S6. Reports excluded at full-text assessment, with reasons

All nine reports assessed at full text and excluded are listed. Every exclusion was made under the same criterion: levetiracetam exposure or the biomarker result could not be separated from pooled multidrug or drug-class data. No report was excluded at this stage for being non-human, in vitro or ex vivo. ASM, antiseizure medication; BAP, biological antioxidant potential; d-ROM, derivatives of reactive oxygen metabolites; LEV, levetiracetam; TBARS, thiobarbituric acid reactive substances.

Report	Journal	DOI	Reason for exclusion
Ersan et al. 2020	Epilepsy Research	10.1016/j.eplepsyres.2020.106352	Only a pooled intervention group (LEV, valproate and carbamazepine combined; n=45) versus controls. The LEV subgroup (n=17) has no separately reported biomarker values, only the statement that markers did not differ between drug types.
Morimoto et al. 2017	Journal of the Chinese Medical Association	10.1016/j.jcma.2016.10.005	d-ROM and BAP reported pooled for lamotrigine plus LEV; all participants on polytherapy (3–4 ASMs, most often with concomitant valproate); oxidative markers not broken down by drug.
Calik et al. 2014	Epilepsy Research	10.1016/j.eplepsyres.2014.08.007	Intractable epilepsy, all participants on multiple ASMs; results stratified only by the number of ASMs (2, 3 or 4), not by drug; no LEV subgroup and no LEV-specific values.
Szołtysek-Bołdys et al. 2025	Brain Sciences	10.3390/brainsci15060655	Polytherapy cohort; the only relevant subgroup is valproate plus LEV (n=6), in which any LEV effect is confounded with valproate; no LEV monotherapy subgroup and no separately reported LEV values.
Menon et al. 2014	Annals of Indian Academy of Neurology	10.4103/0972-2327.144008	The table of individual ASMs reports only carbamazepine, phenytoin and valproate; LEV falls within an “others” category (13%) without drug-specific values.

Medina et al. 2016	RSC Advances	10.1039/c6ra15777g	LEV users (11 of 15) included in a pooled "new-generation ASM" group; neuroprostanes and F2-dihomo-isoprostanes reported by group only, without separate LEV values.
Sarecka-Hujar et al. 2022	Brain Sciences	10.3390/brainsci12010120	Epilepsy on polytherapy (≥2 ASMs) versus controls; although most participants received LEV in combination, no LEV-specific subgroup or analysis is presented.
Tan et al. 2009	Epilepsia	10.1111/j.1528-1167.2009.02024.x	LEV users (n=18) not analysed separately; TBARS and thiols compared only across enzyme-inducer, enzyme-inhibitor and polytherapy groups.
Kopan et al. 2022	International Journal of Neuroscience	10.1080/00207454.2022.2140426	Monotherapy analysed as a single group (valproate, carbamazepine, LEV and lamotrigine combined) versus polytherapy versus controls; no LEV subgroup.

Table S7. Database of retrieval for each included study

Compiled from the review's screening log. A tick indicates that the record was retrieved by that source in the searches of 15 July 2026. It documents how far Scopus and Web of Science cover the included studies, given that Embase was not searched.

Study	PubMed/MEDLINE	Cochrane Library	Scopus	Web of Science	Retrieved by one source only
Varoglu 2010	Yes	Yes	Yes	Yes	No
Ozden 2010	Yes	No	Yes	Yes	No
Chen 2015	Yes	No	Yes	Yes	No
Elsayed 2016	No	No	Yes	No	Yes (Scopus)
Haznedar 2019	Yes	No	Yes	Yes	No
Çoban Ramazan 2019	No	No	Yes	Yes	No
Sarangi 2019	Yes	No	Yes	Yes	No
Mahdavi 2020	Yes	No	Yes	Yes	No
Mehvari-Habibabadi 2022	No	No	Yes	Yes	No
Swathi and Aruna 2022	No	Yes	No	No	Yes (Cochrane)
Wang 2025	No	No	No	Yes	Yes (Web of Science)
Total retrieved (of 11)	6	2	9	9	3

No single database retrieved more than 9 of the 11 included studies. PubMed contributed no study that was not also retrieved by Scopus and Web of Science. Three studies were retrieved by one source only. The review authors therefore do not claim that Scopus and Web of Science compensate for the absence of Embase; these data indicate the opposite, and the omission is reported as a material limitation in Section 4.6 of the manuscript.

Table S8. Target-trial specifications for the three comparator streams

Element	Stream A (within-person change)	Stream B (LEV vs healthy controls)	Stream C (LEV vs another ASM)
Eligibility	People with epilepsy who are candidates for starting LEV	Not definable: healthy volunteers are not eligible for the intervention	People with newly diagnosed epilepsy who are candidates for either drug
Assignment	Random allocation to start LEV or not to start it	No assignment is conceivable between the compared groups	Random allocation to LEV or to a specified alternative ASM
Comparator	Concurrent untreated epilepsy group followed over the same interval	None	A single named alternative antiseizure medication
Outcome	Prespecified redox biomarker measured at baseline and after an identical interval in both arms	Prespecified redox biomarker	Prespecified redox biomarker measured identically in both arms
How included studies depart	No counterfactual arm at all; time, seizure control and regression to the mean are unobservable rather than merely uncontrolled	The contrast estimates the joint effect of having epilepsy and being treated for it	Non-random allocation; in one study the comparator pools three drugs
Consequence for assessment	Serious risk of bias for confounding; see Note S1 on the critical-risk question	Downgraded for indirectness rather than treated as a confounded treatment effect	Serious risk of bias for confounding by indication

Note S1. Target trials and the rationale for not applying a critical risk-of-bias judgment

ROBINS-I requires each non-randomized study to be assessed against a specified target trial. The three target trials used in this review are given in Table S8. Stream B is the exception: because healthy volunteers are not eligible for levetiracetam, no randomized trial corresponds to that contrast, and Stream B is therefore treated as descriptive evidence about treated epilepsy and downgraded for indirectness rather than reported as a confounded estimate of a treatment effect.

For the uncontrolled before-after studies of Stream A we considered whether confounding warranted a judgment of critical rather than serious risk of bias. ROBINS-I reserves 'critical' for a study that is too problematic to provide any useful evidence on the effect of the intervention, and the convention that follows is that such studies are not included in the synthesis at all.

We did not apply it, and we set out the reasoning so that readers may disagree with it. These studies are uninformative about the causal effect of levetiracetam, and the manuscript says so in the Results, the Discussion and the Conclusions rather than leaving it to the risk-of-bias table. What they do provide is a set of verifiable biomarker values measured in defined cohorts, together with a record of how those values were reported. That descriptive content is the material from which this review draws its central claim, namely that the literature cannot answer the question. Rating these studies critical and excluding them would remove the evidence whose limitations the review exists to characterize, and would leave a review of four studies asserting the same conclusion with less support for it.

Readers who prefer the stricter reading may treat Stream A as contributing no causal evidence whatever. No conclusion of this review changes on that reading: every Stream A body of evidence is rated very low certainty either way, no Stream A result is used to support a directional claim, and the sensitivity analysis of Section 3.9 already demonstrates that removing a Stream A study leaves the conclusions intact.

Note S2. Operationalization of the source-verifiability and reporting-consistency check

This check was added after protocol registration and is reported as post hoc. Its purpose is narrow: to identify reports whose content cannot be reconciled with what the report states about itself, so that such a report does not silently become the sole basis of a body of evidence. It is not a research-integrity investigation, it makes no finding about the conduct of any study or the intentions of any author, and it implies none. Every criterion is verifiable by any reader of the published article.

Criterion	Level	Question asked	Answer recorded
(i) Principal method	2	Does the principal method named in the title or abstract appear in the Methods, and does it generate results that are reported?	Yes / No
(ii) Mechanistic attribution	2	Are pharmacological or mechanistic statements attributed to the drug they concern?	Yes / No
(iii) Numerical availability	1	Are numerical results for the review's outcomes tabulated, rather than presented only in figures?	Yes / No
(iv) Dispersion measure	1	Is the dispersion measure identified, and used consistently throughout the report?	Yes / No
(v) Reference handling	2	Is the reference list free of artefacts of automated database parsing?	Yes / No

Level 1 comprises ordinary reporting deficiencies. These are common in this literature — figure-only reporting affects two included studies and inconsistent dispersion reporting affects one — and carry no implication beyond incomplete reporting. They are handled through ROBINS-I domain 7 and through GRADE.

Level 2 comprises unresolved discrepancies between what a report states about itself and what it contains. Four consequences of a Level 2 result were fixed before the check was applied: (a) the report remains in the descriptive synthesis; (b) it cannot be the sole support of a GRADE body of evidence; (c) it does not contribute to judgments of consistency across studies, because consistency presupposes comparable reporting; and (d) it is removed in the sensitivity analysis reported in Section 3.9 of the manuscript.

The check was applied to all eleven included reports, and the result for each criterion is recorded here so that any reader can reproduce it. Criteria (i), (ii) and (v), which define Level 2, were met by ten reports and not by Wang et al. 2025, which is therefore the only report with a Level 2 result. Criterion (iii) was met by nine reports and not by Haznedar et al. 2019 or Wang et al. 2025, whose review-relevant outcomes are presented only in figures. Criterion (iv) was met by ten reports and not by Wang et al. 2025. The authors of that report and the publishing journal were not contacted, for two reasons stated here for transparency: the observations concern what is verifiable in the published text and require no clarification to be verified, and correspondence of that kind is a post-publication process independent of, and on a different timescale from, this review.

Note S3. Handling of linked companion records

Two records retrieved by the searches were linked to an included report rather than being independent studies. Neither was removed on the basis of title similarity. Each was retrieved in full, read against its primary publication, and checked for participants, outcomes or time points not present in that publication.

Record	Source	Linked to	What it contained	Decision
Report of urinary 15-F2t-isoprostane levels in epileptic patients, 2009	Web of Science	Ozden et al. 2010	An earlier report of the same cohort and the same outcome; no participants, outcomes or time points additional to the 2010 publication	Excluded as a companion record; no data carried forward
Trial registry entry, 2017	Cochrane Library	Swathi and Aruna 2022	The registered outcome set for the trial reported in the 2022 publication	Excluded as a companion record; the registered outcome set was carried into the risk-of-bias assessment

The registry entry linked to Swathi and Aruna is the reason that study is the single exception in the review's assessment of selective reporting: it is the only included study for which a prespecified outcome set could be consulted. The registered outcomes were compared with the publication and no measured biomarker was found to be unreported. For the other ten studies, selective reporting could not be excluded.
